# A systematic study towards evolutionary and epidemiological dynamics of currently predominant H5 highly pathogenic avian influenza viruses in Vietnam

**DOI:** 10.1038/s41598-019-42638-4

**Published:** 2019-05-22

**Authors:** Lam Thanh Nguyen, Simon M. Firestone, Mark A. Stevenson, Neil D. Young, Leslie D. Sims, Duc Huy Chu, Tien Ngoc Nguyen, Long Van Nguyen, Tung Thanh Le, Hung Van Nguyen, Hung Nam Nguyen, Tien Ngoc Tien, Tho Dang Nguyen, Bich Ngoc Tran, Keita Matsuno, Masatoshi Okamatsu, Hiroshi Kida, Yoshihiro Sakoda

**Affiliations:** 1 Laboratory of Microbiology, Faculty of Veterinary Medicine, Hokkaido University, Sapporo, Hokkaido, 060-0818 Japan; 2 Department of Veterinary Medicine, College of Agriculture, Can Tho University, Can Tho, Vietnam; 3 Melbourne Veterinary School, Faculty of Veterinary and Agricultural Sciences, The University of Melbourne, Parkville, Victoria, 3010 Australia; 4 Asia Pacific Veterinary Information Services, Montmorency, Victoria, 3094 Australia; 5 Department and Sub-Departments of Animal Health, Ministry of Agriculture and Rural Development, Ha Noi, Vietnam; 6 Regional Animal Health Office VII, Department of Animal Health, Ministry of Agriculture and Rural Development, Can Tho, Vietnam; 7 National Center for Veterinary Diagnostics, Department of Animal Health, Ministry of Agriculture and Rural Development, Ha Noi, Vietnam; 8 Global Institution for Collaborative Research and Education, Hokkaido University, Sapporo, Hokkaido, 001-0020 Japan; 9 Research Center for Zoonosis Control, Hokkaido University, Sapporo, Hokkaido, 001-0020 Japan

**Keywords:** Phylogenetics, Influenza virus

## Abstract

This study aimed to elucidate virus, host and environmental dynamics of Vietnamese H5 highly pathogenic avian influenza viruses (HPAIVs) during 2014–2017. Epidemiologically, H5 HPAIVs were frequently detected in apparently healthy domestic and Muscovy ducks and therefore these are preferred species for H5 HPAIV detection in active surveillance. Virologically, clade 2.3.2.1c and 2.3.4.4 H5 HPAIVs were predominant and exhibited distinct phylogeographic evolution. Clade 2.3.2.1c viruses clustered phylogenetically in North, Central and South regions, whilst clade 2.3.4.4 viruses only detected in North and Central regions formed small groups. These viruses underwent diverse reassortment with existence of at least 12 genotypes and retained typical avian-specific motifs. These H5 HPAIVs exhibited large antigenic distance from progenitor viruses and commercial vaccines currently used in poultry. Bayesian phylodynamic analysis inferred that clade 2.3.2.1c viruses detected during 2014–2017 were likely descended from homologous clade viruses imported to Vietnam previously and/or preexisting Chinese viruses during 2012–2013. Vietnamese clade 2.3.4.4 viruses closely shared genetic traits with contemporary foreign spillovers, suggesting that there existed multiple transboundary virus dispersals to Vietnam. This study provides insights into the evolution of Vietnamese H5 HPAIVs and highlights the necessity of strengthening control measures such as, preventive surveillance and poultry vaccination.

## Introduction

In 2001, H5N1 subtype highly pathogenic avian influenza viruses (HPAIVs), of the A/goose/Guangdong/1/1996 (Gs/GD) lineage, were detected for the first time in geese in Vietnam^1^. By December 2003, other Gs/GD-lineage H5N1 viruses had been detected in the North of Vietnam and by end-2004 had caused large poultry outbreaks in 57 out of 64 provinces^1–3^. Since then, H5 HPAIVs have remained enzootic and primary causative agents of several thousand outbreaks in poultry^4,5^. In addition, Gs/GD-lineage H5 HPAIVs have posed great public health concerns. To date, 127 H5 HPAIV clinical cases have been reported in Vietnam of which 64 were fatal (approximately 50% fatality rate)^6^.

H5 HPAIVs have diversified into multiple clades that are based on genetic variation of the gene encoding the hemagglutinin (HA) protein. The large genetic diversity of Vietnamese Gs/GD-lineage H5 HPAIVs is the result of multiple virus introductions and divergence of viruses circulating domestically^2,3^. The clade 1 H5 HPAIVs, the main cause of outbreaks in Vietnam during 2003–2005, spread from North to Central and South of Vietnam and became enzootic in the entire country. After 2006, clade 1 H5 HPAIVs in the North and Central regions were replaced by other imported Gs/GD-lineage viruses such as clade 2.3.2 in 2005–2008 and 2.3.4 in 2007–2010. Clade 1 viruses persisted in the South region and evolved to produce clade 1.1.1 and 1.1.2 variants^2,7,8^. Besides, several H5 HPAIVs of the clade 5, 7 and 8 viruses were sporadically detected in Vietnam^2^. Since 2013, clade 1.1.2, 2.3.2.1c and 2.3.4.4 H5 HPAIVs have been concurrently predominant in Vietnam^2,9^. Due to persistent circulation of various H5 HPAIVs in Vietnam and the broader regions, reassortment events resulted from exchanging gene segments between H5 HPAIVs and other avian influenza viruses (AIVs) increases genomic diversification. Approximately 56 distinct genotypes, ranging from VN1–VN56, of Vietnamese H5 HPAIVs have been identified^7–10^.

To reduce virus loads in poultry and human health risk, mass poultry vaccination against H5 HPAIVs was implemented in Vietnam in mid-2005^11,12^. Vaccination was followed by a 12 month period in which no new outbreaks in poultry were reported and 18 months before a human case was reported^11,13,14^. Currently, two commercial vaccines are used in poultry vaccination programs. The first contains a clade 1 antigen which is produced from inactivated split-virion A/Vietnam/1194/2004 (H5N1). The second is based on a clade 2.3.2 antigen which produced from inactivated rgA/duck/Guangdong/S1322/2010 (H5N1)^5^. Updating of vaccine antigens has not been performed since the clade 2.3.2 vaccine was introduced^2^. Though mass vaccination has been implemented, it was never anticipated that vaccination would eliminate Gs/GD-lineage H5 HPAIVs from Vietnam. Vaccine failure can occur by several factors including insufficient doses, concurrent immunosuppressive disease and antigenic variants^13^. Experimentally, poultry vaccination accelerates selection of antigenic variants of H5 HPAIVs^15^. In addition, novel strains of Gs/GD-lineage H5 HPAIVs that have a poor antigenic match with existing vaccines have been imported to Vietnam^2^. From a general epidemiological perspective, H5 HPAIV persistence is facilitated by agro-ecological condition in which mobile free-ranging ducks, low biosecurity of backyard/small-scale production sectors and poorly bio-secure live bird markets (LBMs) are all considered as key risk factors^16–18^. Thus, comprehensive knowledge of viral, host and environmental factors affecting H5 HPAIV evolution is essential for successful control and prevention of H5 HPAIVs. Therefore, this study aimed to investigate virological and epidemiological features of contemporary Vietnamese H5 HPAIVs for the 2014–2017 period, using the results of our active surveillance, global surveillance data and longitudinal literature review.

## Results

### Surveillance programs and identifying specific hosts of H5 HPAIVs

**Figure 1:**
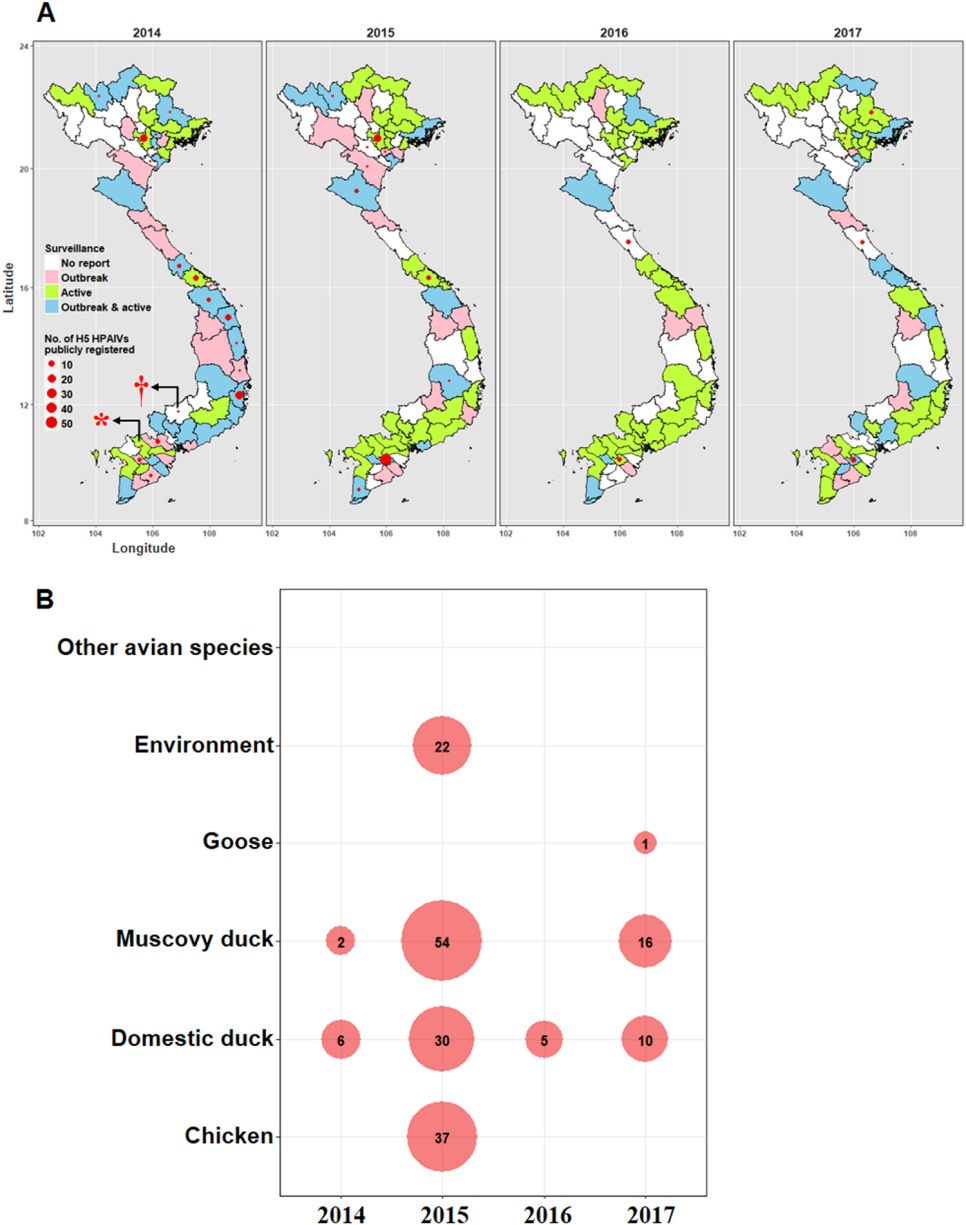
Passive and active surveillance and identifying specific hosts of H5 HPAIVs. (**A**) Space-time distribution of provinces reporting H5 HPAIV outbreaks and active surveillance programs and the number of H5 HPAIV strains publicly registered (GISAID and/or IRD) in given provinces during 2014–2017. Province centroids are used to localize total number of H5 HPAIV strains published in each province. * and † indicate H5 HPAIVs detected from human cases of clade 1.1.2 and 2.3.2.1c viruses, respectively. (**B**) Summary statistics of domestic poultry species and environmental samples positive for H5 HPAIVs in our surveillance program. Number and size of each circle indicates sum of infected individuals.

Profiles gathered from passive and active surveillance programs in poultry in Vietnam were reviewed. The choropleth map in Fig. 1A illustrated space-time distribution of provinces reporting H5 HPAIV outbreaks as denoted via passive notification and provinces conducting active surveillance for the period 2014–2017. Outbreaks of disease associated with H5 HPAIVs were reported in all regions (North, Central and South) in each year, indicating that H5 HPAIVs persisted or occurred in a wide geographic and temporal range. Some variation in the number of provinces reporting outbreaks was apparent, ranging from 8 in 2016 to 35 in 2014 (Fig. 1A and Supplementary Table S1).

Active surveillance programs were deployed in all three regions of Vietnam. The number of H5 HPAIV strains detected in each province that were available in public influenza virus databases were co-plotted in the choropleth map. Within the 4-year period, a substantial number of Vietnamese H5 HPAIV strains (n = 192) were deposited in the public databases. More viruses were deposited in 2014 compared to 2016 in line with the increase in the number of outbreaks (Fig. 1A). These results demonstrate the importance of surveillance for monitoring H5 HPAIVs in Vietnam and its contribution to global influenza surveillance.

Regarding our work, 183 H5 HPAIVs were isolated from 12,585 swab samples from our surveillance program (Supplementary Table S2). Of these, 77 geographically and temporally representative isolates were selected for sequencing (Supplementary Table S3). The results from virus isolation in our active surveillance of apparently healthy birds showed that domestic ducks (51 isolates) and Muscovy ducks (72 isolates) were the species from which H5 HPAIVs were most frequently isolated (Fig. 1B). Likewise, the number of infected birds with other Vietnamese H5 HPAIV strains deposited in global surveillance databases (unknown whether passive or active sources) showed a similar pattern (Supplementary Fig. S1). Therefore, domestic and Muscovy ducks are preferred species for detection of H5 HPAIVs in active surveillance.

### Distinct phylogeography and genomic diversity of contemporary Vietnamese H5 HPAIVs

**Figure 2:**
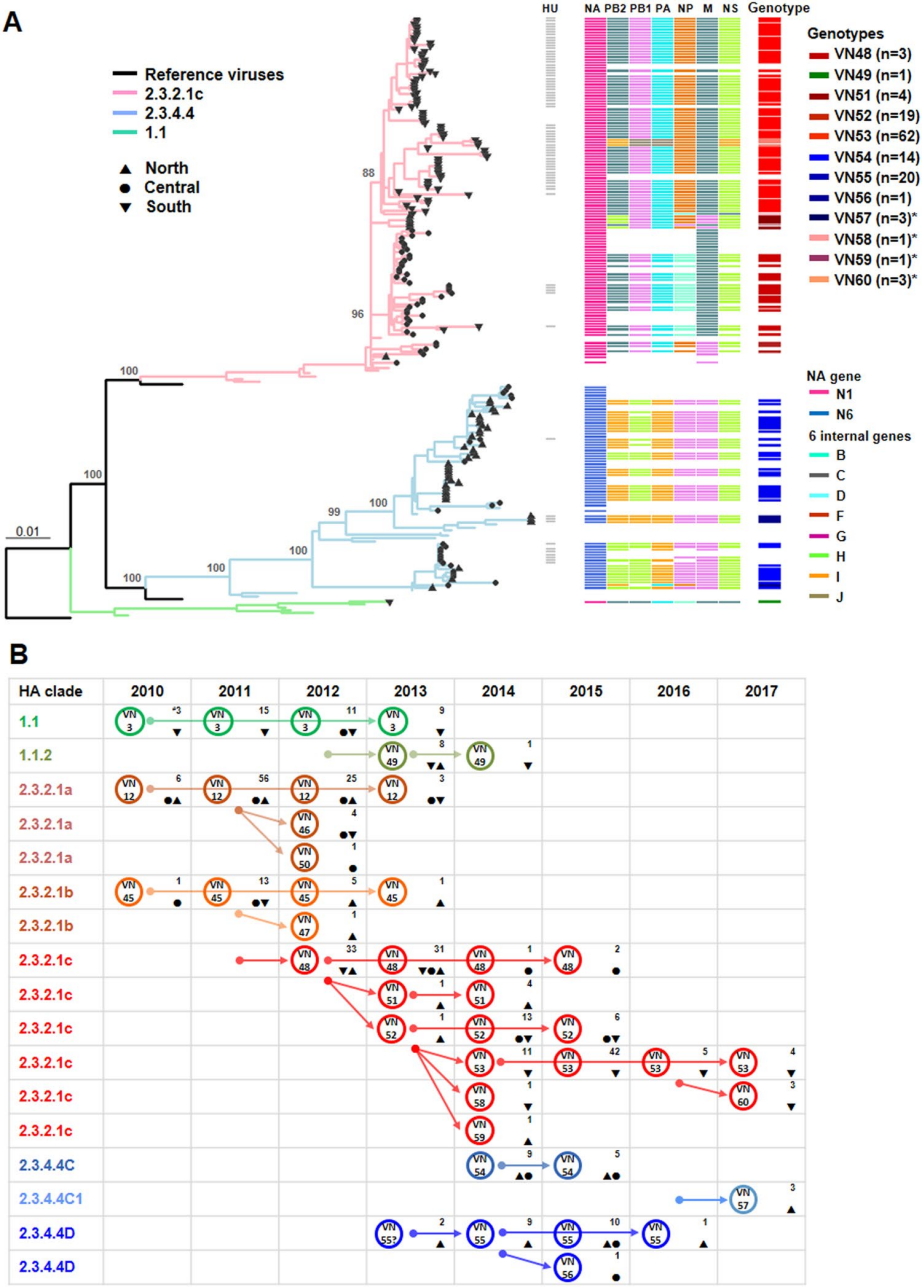
Phylogeography and genomic diversity of contemporary H5 HPAIVs. (**A**) Maximum likelihood phylogenetic tree of H5 HA gene segments (left panel) and viral genotypes (right panel) of Vietnamese H5 HPAIVs during 2014–2017. Viruses isolated by our Hokkaido University (HU) surveillance program are indicated in corresponding horizontal gray lines (middle column panel). Supplementary Fig. S2A shows annotated tree with full strain names. Bootstrap values are shown for key nodes. The total (n) of H5 HPAIV strains per genotype is indicated in parentheses. Asterisks (*) indicate newly identified genotypes in this study. For detailed color interpretation, readers are advised to refer the online version of this article. (**B**) Schematic space-time genotypic transition of Vietnamese H5 HPAIVs from 2010 to 2017. Genotypes are indicated inside circles. Number (#) of H5 HPAIV strains of each genotype per year is indicated in each square. Arrows indicate the probable transition of each genotype following the existence of previous homologous HA clade assignment. Geographic origins of each virus/genotype are shape-coded as North (▲), Central (●) and South (▼) regions.

To determine clade and genotype designation of Vietnamese H5 HPAIVs, RNA sequences of the 77 representative viruses from our surveillance study were combined with H5 HPAIV sequence data deposited in the global influenza virus databases between 2014 and 2017. The maximum likelihood (ML) phylogenetic tree of H5 HA genes constructed from this combined dataset showed that predominant H5 HPAIVs during 2014–2017 belonged to the 2.3.2.1c and 2.3.4.4 HA clades. Only one strain in the clade 1.1.2 was reported from a human case in 2014 (Fig. 2A and Supplementary Fig. S2A). It was evident that clade 2.3.2.1c and 2.3.4.4 viruses formed distinct phylogenetic and geographic groups. Clade 2.3.2.1c viruses were detected in all three North, Central and South regions and with few exceptions clustered phylogenetically within each region. Indeed, clade 2.3.2.1c viruses circulating in the South formed an independent cluster from 2.3.2.1c viruses in the North and Central regions. On the other hand, clade 2.3.4.4 viruses only reported in the North and Central regions formed two distinct clusters within clade 2.3.4.4, previously assigned as 2.3.4.4C and 2.3.4.4D^19,20^ (the provisional nomenclature scheme was referred for the purpose of this study only) (Supplementary Fig. S2A). In 2017, clade 2.3.4.4C H5 HPAIVs were also detected and these viruses formed phylogenetically distinct from progenitor clade 2.3.4.4C viruses (100% nodal support). We have referred these as the clade 2.3.4.4 group C1 (2.3.4.4C1) and that met the WHO/OIE/FAO standard nomenclature criteria^21^ (Supplementary Fig. S2A). Similarly, phylogenetic relationships were observed for the NA gene segment. N1 NA genes were inferred to be descendants of N1 genes of H5N1 HPAIVs that previously existed in Vietnam and mostly grouped by the three geographic regions (Supplementary Fig. S2B). The N6 NA genes formed into three distinct groups following the clade assignments of HA gene segment, 2.3.4.4C, 2.3.4.4C1 and 2.3.4.4D (Supplementary Fig. S2C).

Phylogeny of the other six internal gene segments were assigned following previous systems^7–10^, each gene segment (i.e., PB2, PA, NP, M or NS) formed into 2–3 clusters (Supplementary Fig. S2D,F–I). PB1 genes were likely more diverse and formed four distinct clusters in the phylogenetic tree (Supplementary Fig. S2E). In addition, we identified novel gene cluster(s) in the PB2, PB1, PA, NP and NS gene segments (Supplementary Fig. S2D–G,I).

On the basis of viral reassortment capacity, a total of 132 H5 HPAIV strains (63 strains from our surveillance), which have full genome sequences, were grouped into 12 genotypes, four of which were identified for the first time in this study (Fig. 2A and Supplementary Table S5). Clade 2.3.2.1c viruses had a greater number of genotypes (n = 7) compared to the number of genotypes in clade 2.3.4.4 (n = 4). The result indicates that Vietnamese H5 HPAIVs have continued its diverse genomic evolution although it is not clear whether these viruses have reassorted in Vietnam or elsewhere.

To study genotypic transition of Vietnamese H5 HPAIVs, genotype identification from previous studies for the period 2010–2013^9,10^ was collated in our analysis. Figure 2B schematically represents spatial-temporal genotypic dynamics of Vietnamese H5 HPAIVs from 2010 to 2017. Overall, genotypes VN48, VN52, VN53 and VN60 of clade 2.3.2.1c viruses were identified in recent years. The VN54, VN55 and VN57 genotypes of clade 2.3.4.4 viruses concurrently existed in the North and Central regions. Several transient genotypes (VN46, VN47, VN49, VN50, VN51, VN56, VN58 and VN59) were also identified. Furthermore, temporal likelihood of genotype replacement was observed in 2012–2013. Since the VN3 genotype of clade 1.1 viruses and the VN12, VN45, VN46, VN47 and VN50 genotypes of clade 2.3.2.1c viruses predominantly co-circulated in earlier years, but had disappeared by 2013. Conversely, clade 2.3.4.4 viruses also first emerged in 2013 and became dominant with several genotypes (Fig. 2B). These results reveal that Vietnamese H5 HPAIVs have evolved with considerable genetic diversity and replacement of genotypes and clades over time.

### Molecular characterization of the two predominant clade H5 HPAIVs

Conceptually translated viral protein sequences of clade 2.3.2.1c and 2.3.4.4 H5 HPAIVs in Vietnam were characterized to examine genotypic changes when compared to the Gs/GD, the parent of Gs/GD-lineage H5 HPAIVs. For the HA protein sequences, both clade 2.3.2.1c and 2.3.4.4 viruses had four cleavage site motifs containing multi-basic amino acids and all of the motifs differed from the Gs/GD motif which had an additional basic amino acid (Table 1). The majority of clade 2.3.2.1c and 2.3.4.4 H5 HPAIVs either harbored the cleavage site motif QRRRRKR↓G (90.2%) or LRRRRKR↓G (70.7%), respectively. Receptor binding sites in the HA were examined to identify host-specific receptor binding preferences. All of clade 2.3.2.1c and 2.3.4.4 H5 HPAIVs retained avian-type receptor binding signatures, resembling to the Gs/GD virus. All strains examined had residues of 190E, 225G, 226Q and 228G (note: mature H3 HA numbering is used throughout) in the 190-helix and 220-loop, respectively (except for the 130-loop motif). Similarly, almost all of the H5 HPAIV strains did not possess any known mutation for potential mammalian adaptation at positions 627, 701 and 526 in the PB2. Considerable molecular change between the Gs/GD and recent Vietnamese H5 HPAIVs was found in the NA protein stalk truncation. All of clade 2.3.2.1c viruses had 20 amino acid deletions in the N1 NA protein as compared to N1 NA of the Gs/GD (N1 numbering). On the other hand, 36% of clade 2.3.4.4 viruses had full length N6 NA, 470 amino acids similar to the A/environment/Zhenjiang/C13/2013 (H5N6)^22^. The remaining 64% of the viruses lacked 11 amino acids in their stalk region (N6 numbering). It has been demonstrated that NA stalk truncation has arisen from evolutionary adaptation of H5 HPAIVs from wild aquatic birds to terrestrial poultry. Therefore, it might be concluded that recent Vietnamese H5 HPAIVs have retained typical avian-specific motifs and high adaptability in terrestrial avian species although most of these viruses were isolated from aquatic poultry.

**Table 1:** Molecular characterization of the viral proteins of predominant H5 HPAIVs isolated in Vietnam during 2014–2017.

Viral protein	Phenotype	Gs/GD motif^a^	Amino acid motifs (%)^b^		Function
			2.3.2.1c H5 HPAIVs	2.3.4.4 H5 HPAIVs	
HA	Cleavage site	QRRRRKKR↓G	QRRRRK-R↓G (90.2) QRRRKK-R↓G (4.0) QKRRRK-R↓G (2.9) QRRRRR-R↓G (2.9)	LRRRRK-R↓GLF (70.7) LRKRRK-R↓GLF (23.1) LRRRKK-R↓GLF (4.6) LRKRRR-R↓GLF (1.6)	Signature of HPAIVs
	130-loop	130-DASS-133	130-EASL-133 (100.0)	130-ETSL-133 (93.9) 130-ETSS-133 (4.6) 130-EASL-133 (1.5)	Receptor binding pocket
	190-helix	190E	190E (100.0)	190E (100.0)	Receptor binding pocket
	220-loop	225G 226Q 228G	225G (100.0) 226Q (100.0) 228G (100.0)	225G (100.0) 226Q (100.0) 228G (100.0)	Receptor binding pocket
	Antigenic region A	124P, 125S	124K, 125D (87.7) 124K, 125N (9.7) 124K, 125G (2.6)	124K, 125S (100.0)	Antigenic relatedness
		144R, 145S	144N, 145S (96.7) 144N, 145P (3.3)	144M , 145P (70.1) 144T , 145P (24.6) 144V , 145P (4.6)	Antigenic relatedness
	Antigenic region B	128S, 129N	128S , 129D (83.7) 128S , 129N (14.6) 128P , 129D (0.8) 128L , 129D (0.8)	128P , 128N (64.6) 128T , 128N (17.8) 128S , 128N (7.7)	Antigenic relatedness
	Antigenic region B	158N, 159S	158N, 159N (58.5) 158D, 159N (38.2) 158N, 159D (3.3)	158N, 159D (100.0)	Antigenic relatedness
	Antigenic region B	166R, 167S	166K, 167G (87.8) 166K, 167D (8.1) 166K, 167S (4.1)	166M , 167S (67.6) 166I , 167S (26.2) 166V , 167S (6.2)	Antigenic relatedness
	Antigenic region B	187D, 188B	187D, 188E (97.6) 187D, 188G (1.6) 187N, 188E (0.8)	187N, 188A (53.8) 187N, 188E (36.9) 187D, 188A (7.5) 187N, 188G (1.5)	Antigenic relatedness
	Antigenic region B	193K, 194L	193R , 194L (92.6) 193K , 194L (7.4)	193N , 194L (56.9) 193S , 194I (33.8) 193D , 194L (6.2) 193N , 194I (1.5) 193S , 194L (1.5)	Antigenic relatedness
	Antigenic region C	271L, 272E	271V , 272E (68.3) 271V , 272K (30.0) 271M , 272E (1.7)	271M , 272E (60.0) 271V , 272E (26.2) 271I , 272E (13.8)	Antigenic relatedness
NA	N1 stalk deletion^c^	No deletion	50-69del (100.0)	N/A	Enhanced pathogenicity in chickens
	N6 stalk deletion^d^	No deletion	N/A	59-69del (64.0) No deletion (36.0)	Enhanced pathogenicity in chickens
	E119A/G	119E	119E (100.0)	119E (100.0)	Neuraminidase inhibitor resistance
	I222M	222I	222I (100.0)	222I (100.0)	Neuraminidase inhibitor resistance
	R292K	292R	292R (100.0)	292R (100.0)	Neuraminidase inhibitor resistance
	R371K	R371	R371 (100.0)	R371 (100.0)	Neuraminidase inhibitor resistance
PB2	E627K	627E	627E (100.0)	627E (100.0)	Enhanced polymerase activity and virulence in mammalian
	D701N	701D	701D (100.0)	701D (100.0)	Enhanced polymerase activity and virulence in mammalian
	K526R	526K	526K (98.2) 526R (1.8)	526K (100.0)	Enhances the function of 627K and 701N
M2	N31S	31S	31S (100.0)	31S (100.0)	Reduced susceptibility to amantadine/rimantadine

^a^Amino acid motif of the A/goose/Guangdong/1/1996 (H5N1). ^b^Highly variable sites in the antigenic regions of HA are underlined. ^c^N1 NA of A/goose/Guangdong/1/1996 (H5N1) is referred. ^d^N6 NA of A/environment/Zhenjiang/C13/2013 (H5N6) is referred. N/A: not available.

Residues in the putative antigenic regions in the HA were highly variable. Antigenic region B had more mutated sites than the A, C, D and E regions (Table 1). Particularly, positions with highly frequent mutations were found at the region A: 125, 144 and 145; region B: 128, 129, 158, 159, 166, 167, 188 and 193; and region C: 271. These highly variable sites were presumably associated with antigenic variation of H5 HPAIVs.

### Antigenic diversity of H5 HPAIVs and antibody titers of field vaccinated poultry antisera

Antigenicity of Vietnamese H5 HPAIVs representing all clades and subclades detected in this study was characterized using cross hemagglutination inhibition (HI) test (Table 2). Homologous HI titer of the clade 1.1 virus was 1,024 HI, which demonstrated 32–256 fold higher titers compared to viruses from the clade 2.3.4.4 and 2.3.2.1c. Similarly, the homologous titer of the old 2.3.2.1c (Dk/VN/2202/2012) virus was 1,024 HI, which was 4–256 fold higher compared to the heterologous clade viruses and 4–16 folds greater than the more recent clade 2.3.2.1c viruses. Antiserum against a recent clade 2.3.2.1c virus, Dk/VN/386/2015, reacted well with other clade 2.3.2.1c viruses isolated from 2015 to 2017 (1–2 fold differences in titers). Antisera against the clade 2.3.4.4 viruses (Ck/Kum/1–7/2014 and Bs/Akt/1/2016) reacted well with other homologous clade 2.3.4.4 viruses but did not react well with clade 2.3.2.1c viruses by a reduction of 4–512 folds. These results demonstrated that contemporary clade 2.3.2.1c and 2.3.4.4 viruses were antigenically distinct from each other and from clade 1.1 viruses.

**Table 2:** Cross-reactivity of plaque-cloned H5 HPAIVs with laboratory antisera panel using HI test. ^a^Viruses isolated in this study are highlighted in bold. ^b^Homologous titers are underlined.

Viruses^a^	Clade/subclade	Antiserum to^b^					
		Mdk/VN/559/11	Dk/VN/2202/12	Dk/VN/386/15	Ck/Kum/1-7/14	Dk/VN/1151/14	Bs/Akt/1/16
A/Muscovy duck/Vietnam/OIE-559/2011 (H5N1)	1.1	1,024	32	512	512	32	64
A/duck/Vietnam/OIE-2202/2012 (H5N1)	2.3.2.1c	32	1,024	1,024	64	32	128
**A/duck/Vietnam/HU3-386/2015** (**H5N1**)	2.3.2.1c	16	256	2,048	256	64	64
**A/chicken/Vietnam/HU4-1328/2015** (**H5N1**)	2.3.2.1c	16	64	512	128	16	16
**A/duck/Vietnam/HU5-1571/2016** (**H5N1**)	2.3.2.1c	16	256	1,024	8	16	16
**A/duck/Vietnam/HU8-1817/2017** (**H5N1**)	2.3.2.1c	16	64	512	8	8	16
A/chicken/Kumamoto/1-7/2014 (H5N8)	2.3.4.4icA	16	16	256	2,048	1,024	256
**A/Muscovy duck/Vietnam/HU2-26/2014** (**H5N6**)	2.3.4.4D	16	8	64	512	1,024	256
**A/duck/Vietnam/HU1-1151/2014** (**H5N6**)	2.3.4.4D	16	8	64	1,024	2,048	512
A/black swan/Akita/1/2016 (H5N6)	2.3.4.4C	16	8	64	256	256	512
**A/chicken/Vietnam/HU4-42/2015** (**H5N6**)	2.3.4.4C	16	16	256	512	512	512
A/duck/Japan/AQ-HE72/2015 (H5N6)	2.3.4.4C	8	8	256	1,024	2,048	512
A/chicken/Japan/AQ-HE144/2015 (H5N6)	2.3.4.4C1	16	8	256	1,024	1,024	512
**A/Muscovy duck/Vietnam/HU7-20/2017** (**H5N6**)	2.3.4.4C1	16	8	256	512	512	512

**Figure 3:**
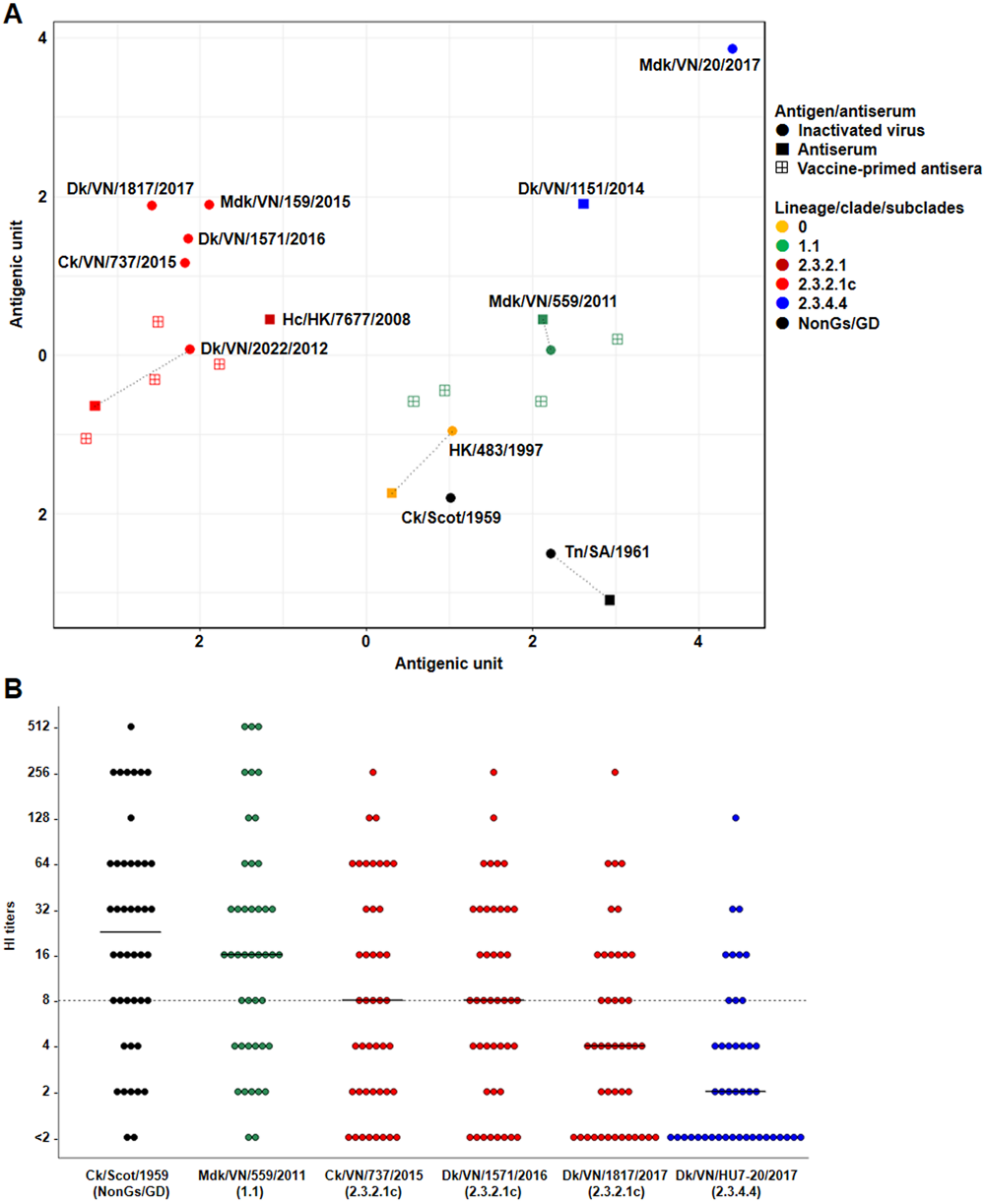
Antigenic evolution of Vietnamese H5 HPAIVs and antibody titers of field vaccinated poultry antisera. (**A**) Antigenic cartography of representative Vietnamese H5 HPAIVs. Both vertical and horizontal axes represent antigenic distance. The spacing between gridlines is equivalent to an antigenic unit distance corresponding to a 2-fold HI difference. The dot lines indicate a combination of the homologous viruses and antisera. Vaccine-primed antisera were prepared in laboratory condition. Supplementary Table S6 describes abbreviation and HI titers. (**B**) Detectable HI titers of 44 vaccinated poultry antisera samples collected in the field against the reference antigen and representative inactivated H5 HPAIVs. The linear black lines indicate the median of antibody levels within each population. The horizontal dot line at 8 HI is the minimum protective level^23^.

To examine the antigenic evolution of Vietnamese H5 HPAIVs, representative viruses were cross-reacted with laboratory reference antisera and antisera against the commercial clade 1 and 2.3.2 vaccines produced in laboratory condition using HI test. The cross-HI titers were used to produce antigenic cartography. Many of the inactivated viruses and homologous antisera formed independent groups with distances of at least approximately two antigenic units (4-fold HI difference) following distribution of each H5 HA gene assignment (Fig. 3A). For instance, the commercial inactivated Ck/Scot/1959 virus, the reference antigen currently used for assessing vaccination coverage in Vietnam, displayed a large antigenic distance from 2.3.2.1c and 2.3.4.4 H5 HPAIVs, regardless of the close antigenic relationship to clade 0 H5 HPAIV (HK/483/1997). Laboratory antisera produced from chickens and ducks immunized with the commercial clade 1 and 2.3.2 vaccines antigenically surrounded the clade 1.1 and 2.3.2.1c H5 HPAIVs of Mdk/VN/559/2011 and Dk/VN/2202/2012, respectively, but were distant from the contemporary 2.3.2.1c and especially 2.3.4.4 viruses. This result confirmed that clade 2.3.4.4 viruses were antigenically different from progenitor H5 HPAIVs, the reference antigen and commercial vaccine antigens currently used in poultry vaccination in Vietnam. Recent clade 2.3.2.1c H5 HPAIVs showed some minor antigenic variation from clade 2.3.2 vaccine and major variation from clade 1 vaccine and the reference antigen. In other words, use of clade 1 and 2.3.2 vaccines would be expected to provide little protection for clade 2.3.4.4 H5 HPAIVs. Updates of vaccine strains and reference antigen that have close antigenic match with recent viruses should be considered for better vaccination programs.

To evaluate antibody titers of vaccinated birds in the field, 60 field antisera samples from vaccinated domestic poultry were tested with the reference antigen and representative inactivated Vietnamese H5 HPAIVs. Detectable titers of the 44 antisera samples were shown in Fig. 3B and Supplementary Table S8. The majority of field antisera exhibited protective HI titers (>8HI)^23^ against the Ck/Scot/1959 reference antigen and the inactivated clade 1.1 Mdk/VN/559/2011 virus. Median HI titers of field antisera against the clade 2.3.2.1c H5 HPAIVs isolated in 2015 and 2016 stayed at 8 HI but gradually reduced 2–4 folds against clade 2.3.2.1c and 2.3.4.4 viruses isolated in 2017, respectively. Taken the above results together, commercial clade 1 vaccine might provide potent protection from earlier viruses but partial protection recently circulating H5 HPAIVs. The clade 2.3.2 vaccine likely remains effective against recent 2.3.2.1c H5 HPAIVs, but effectiveness of the 2.3.2 vaccine against clade 2.3.4.4 viruses was uncertain.

### Distinct spatiotemporal phylodynamics of the two predominant clade H5 HPAIVs

The Bayesian phylogenetic (BP) method was implemented to infer phyloepidemiology of the two predominant viruses via tracing genetic traits of H5 HA gene segments. HA gene sequences of clade 2.3.2.1c and 2.4.4.4 H5 HPAIVs since their first detection in Vietnam and homologous clade viruses in Laos, Cambodia and southern neighboring provinces of China were included in the analysis. Phylogenetically, clustering of the two clade HA segments in the BP trees was highly identical to ML trees on the basis of clade assignment as above described (Supplementary Fig. S3A,B). The BP trees of clade 2.3.2.1c and 2.3.4.4 HA segments had distinct topological patterns. HA gene segments of the Vietnamese clade 2.3.2.1c H5 HPAIVs during 2014–2017 were genetically distinct from the clade 2.3.2.1c viruses detected in China during 2014–2016 (no genetic information of clade 2.3.2.1c virus was reported in China in 2017). HA gene segments of recent 2.3.2.1c Vietnamese viruses were more likely descended from homologous clade viruses previously introduced in Vietnam and/or preexisting Chinese viruses during 2012–2013 (Supplementary Fig. S3A). Differently, the 2.3.4.4 HA genes formed multiple small clusters aggregated with abundant homologous clade viruses circulating concurrently in China. This indicated that for the period 2014–2017, these two predominant clade 2.3.2.1c and 2.3.4.4 viruses in Vietnam had distinct genetic evolution.

**Figure 4:**
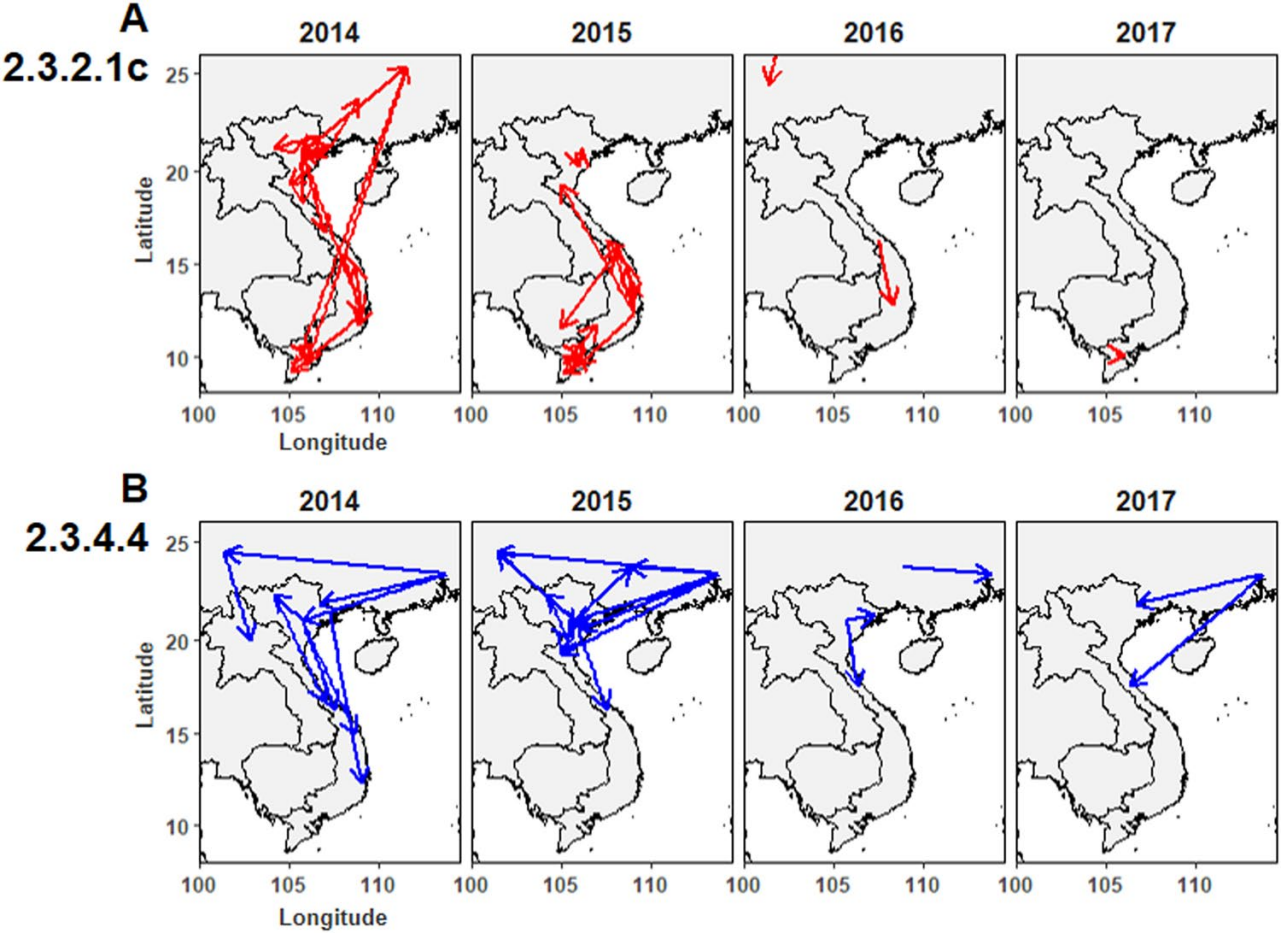
Spatiotemporal phylodynamics of two predominant clade 2.3.2.1c (**A**) and 2.3.4.4 (**B**) H5 HPAIVs in Vietnam and other neighboring countries from 2014 to 2017. Epidemiological dispersal linkages from one location to another are indicated by arrows.

To explore the phylodynamics of these populational viruses further, dispersal traits annotated from the BP trees were spatiotemporally constructed and visualized in Fig. 4. During 2014–2017, incursion of the clade 2.3.2.1c viruses was more likely enclosed within Vietnam and with multiple internal linkages. A few dispersal linkages of clade 2.3.2.1c viruses in Vietnam and other neighboring countries were identified (Fig. 4A). It is noteworthy that clade 2.3.2.1c viruses during 2012–2013, period of their first introduction in Vietnam, had multiple genetic linkages with Chinese viruses (Supplementary Fig. S4A). On the other hand, most of Vietnamese clade 2.3.4.4 viruses in the period of 2014–2017 had multiple genetic linkages with homologous clade 2.3.4.4 viruses currently predominant in China. The first introduction of the clade 2.3.4.4 viruses in 2013 was traceable by a transboundary linkage of highly identical genetic traits to the Guangdong viruses (Supplementary Fig. S4B). Afterward, at least two more introductory linkages were spatiotemporally traced from the southern bordering region of China to northern Vietnam in 2014, 2015 and 2017 (Fig. 4B). Therefore, it might be concluded that emergence/re-emergence of hetero-subclade 2.3.4.4 viruses in Vietnam were consequent from spillovers of foreign viruses. Finally, these results revealed that the predominant Vietnamese 2.3.2.1c and 2.3.4.4 viruses underwent distinct evolution during 2014–2017 and emergence/re-emergence of clade 2.3.4.4 H5 HPAIVs in Vietnam during the study period likely resulted from transboundary spillovers.

## Discussion

Vietnam is regarded as being endemically infected with Gs/GD-lineage H5 HPAIVs. Various control measures have been taken but virus elimination is not regarded as possible in the medium term. Studies aimed to identify viral, host and environmental dynamics of recently circulating H5 HPAIVs are essential to reinforce provisional control measures. The present study was able to systematically elucidate evolutionary and epidemiological dynamics of recent Vietnamese H5 HPAIVs using interdisciplinary analysis.

Regarding the surveillance of H5 HPAIVs in Vietnam, our spatial descriptive analysis illustrated that active and passive surveillance system in Vietnam allowed detection of viruses and disease outbreaks although it also demonstrated that some gaps exist that could result in non-detection of viruses. This work significantly contributed to global influenza surveillance via publishing substantial genetic data of detected viruses (Fig. 1A and Supplementary Table S3). However, it is also critical to discuss some constraints in the system. For instance, in the passive surveillance program, genetic information of H5 HPAIVs that caused outbreaks remained limited. The majority of virus sequences available in public databases were derived from active surveillance programs. A human case infected with a clade 1.1.2 virus was reported in a southern province in 2014, but no outbreak case and genetic information of clade 1.1.2 virus were reported in poultry, posing uncertainty about the actual circulation of viruses in this area (Fig. 1A). These examples emphasize the need to further develop surveillance, particularly virus investigation post surveillance and more focus on high risk areas. In addition, close collaboration between veterinary and human health sectors is needed for effective monitoring and control of AIVs.

In our surveillance, H5 HPAIVs were largely and frequently detected in apparently healthy domestic and Muscovy ducks (Fig. 1B). Role of domestic ducks for H5 HPAIV persistence has been demonstrated^24,25^, but the role of Muscovy ducks remains uncertain. Previous studies have reported that Muscovy ducks were more susceptible to Gs/GD-lineage H5 HPAIVs and vaccination was less effective in preventing infection compared with domestic ducks^26,27^. This result reinforces the epidemiological importance of domestic ducks and justifies further exploration of the role of Muscovy ducks. They remain good targets for active surveillance (Fig. 1B and Supplementary Fig. S1). Moreover, all H5 HPAIVs in our surveillance were isolated in LBMs and LBM-like locations, indicating that LBMs play important roles for virus persistence and dissemination^18,28^. These findings confirmed relatively constant epidemiology of H5 HPAIVs in regards that abundance of waterfowl species and live poultry trading activities facilitate H5 HPAIV persistence and these elements should be primary targets of the surveillance and control system.

In this study, we extended the genetic analysis of H5 HPAIVs to include all Vietnamese viruses that were uploaded in global influenza surveillance databases during the study period. This analysis allowed us to identify novel cluster(s) in the viral segments and elucidate the distinct phylogeography of two predominant clade 2.3.2.1c and 2.3.4.4 viruses (Fig. 2A and Supplementary Fig. S2). Clade 2.3.4.4 viruses likely exhibited greater divergence with the presence of at least three distinct HA subclades comprising the 2.3.4.4C, 2.3.4.4C1 and 2.3.4.4D. The novel subclade 2.3.4.4C1 detected in a border province in the North of Vietnam in 2017 shared a common ancestor with overseas spillovers^29^. Not surprisingly, clade 2.3.2.1c viruses persisted across regions of Vietnam and 2.3.2.1c viruses circulating in the South phylogenetically evolved into an independent population. The similar evolutionary process was also observed for clade 1.1 and its derivatives in the South of Vietnam and Cambodia. This persistent evolution is probably facilitated by the abundance of waterfowl species and some degree of trading segregation of the South^24,25^.

In addition to high variability of the HA gene segment, the entire genomic backgrounds of H5 HPAIVs were divergent. A total of four novel genotypes out of 12 circulating genotypes were identified during the period 2014–2017 (Fig. 2A). Despite multiple reassortment events from exchanges of internal gene segments, only N1 and N6 NA gene segments were consistently assembled with clade 2.3.2.1c and 2.3.4.4 H5 HA segments, generating two high risk H5N1 and H5N6 subtype viruses, respectively. These combinations of the two surface proteins HA/NA probably affords a fitness advantage for H5 HPAIV persistence in Vietnam. From our longitudinal review analysis, genotype transition of Vietnamese H5 HPAIVs was highly dynamic; several genotypes (VN48, VN52, VN53, VN54 and VN55) frequently emerged and then gained dominance from 2012 to 2017 (Fig. 2B). Once a genotype disappeared it did not tend to reappear, suggesting that genomic transition of Vietnamese Gs/GD-lineage H5 HPAIVs followed continuing process in accordance with existence of the HA assignments.

Molecular characterization via viral protein sequences provides information on likely phenotypic characteristics of viruses. Generally, H5 HPAIVs have remained adapted to domestic terrestrial birds and this was also the case with recent H5 HPAIVs in Vietnam which did not contain any mutation makers associated with mammalian adaptation and/or virulence (Table 1). Nonetheless, the threats to human health are always present; in fact, H5 HPAIVs possessing typical avian phenotypes triggered human infections in 2014 (Fig. 1A)^30–32^. This implies complexity of virological and epidemiological interactions and gaps of our understanding H5 HPAIV human infection in nature.

Our antigenic analysis results were highly consistent with previous studies, indicating that H5 HPAIVs recently detected in Vietnam had considerable antigenic variation^9,33,34^. The two predominant clade 2.3.2.1c and 2.3.4.4 H5 HPAIVs from 2014 to 2017 antigenically differ from each other and from clade 1.1 H5 HPAIVs (Table 2). Importantly, these predominant H5 HPAIVs had a large antigenic distance with progenitor H5 HPAIVs, the reference antigen and clade 1 vaccine. Recent clade 2.3.2.1c H5 HPAIVs also exhibited minor antigenic variation with clade 2.3.2 vaccine currently used in poultry in Vietnam (Fig. 3A). Thus, current vaccines should be reassessed through challenge trials. Clade 1 vaccine was unlikely to provide appropriate protection from disease^9,33,35^. Clade 2.3.2 vaccine is expected to be effective against recent 2.3.2.1c H5 HPAIVs but this needs to be monitored closely as some antigenic drift was apparent. Besides, vaccination assessment should be performed using both vaccine antigens and field strains and old reference antigens such as Ck/Scot/1959 should be changed to match the antigenic characteristics of circulating viruses.

Little is known about actual immunity of vaccinated poultry in the field against circulating viruses. Therefore, we assessed the field antisera of vaccinated poultry with the reference antigen and recent H5 HPAIVs (we have assumed that the titers are the result of vaccination and not field exposure but this possibility needs to be recognized in interpreting the data). Our results were in line with other reports that vaccination achieved sufficient coverage and protective antibody titers against the reference antigen and classical H5 HPAIVs (Fig. 3B). However, vaccination likely reduced effectiveness to recently circulating viruses^35,36^. Supposedly, 2.3.4.4 viruses likely become more outspread due to its greater ability to escape from current vaccine-induced immunity. Therefore, it is necessary to reconsider selection of vaccine strains that have close antigenic match to currently circulating H5 HPAIVs to enhance vaccination effectiveness.

Strikingly, we could pinpoint spatiotemporal phylodynamics of Vietnamese H5 HPAIVs using large-scale sequence datasets of HA gene segments. During 2014–2017, two predominant 2.3.2.1c and 2.3.4.4 viruses had distinct phyloepidemiology. Recent clade 2.3.2.1c H5 HPAIVs more likely evolved from homologous clade viruses that were previously introduced in Vietnam and/or preexisted in China during 2012–2013 and persisted across Vietnam via internal dispersals; whereas emergences of heterologous subclade 2.3.4.4 H5 HPAIVs were due to sporadic spillovers of contemporary Chinese 2.3.4.4 viruses to upper parts of Vietnam (Fig. 4). Much greater genetic linkages of Vietnamese and Chinese clade 2.3.4.4 viruses compared to clade 2.3.2.1c viruses were identified during 2014–2017. This might be due to the fact that 2.3.4.4 H5 HPAIVs recently became dominant and replaced 2.3.2.1c viruses in southern Chinese provinces^37,38^. These results underlined the notion that transboundary epidemiological linkages for virus importation to Vietnam exist. In fact, a live poultry movement network between Vietnam and other neighboring countries, including China and Cambodia likely accelerates divergence of Vietnamese H5 HPAIVs^39^. Our speculation is supported by the notion that emergence/re-emergence of H5 HPAIVs in Vietnam is the primary consequence of transboundary spillovers^2^. Yet, we avoided to deeply interpret outwards dispersal linkages derived from Vietnam since interpretation lacks supportive evidence and carries biases. For instance, we only computed H5 HPAIV strains in several Chinese provinces bordering Vietnam, and inferred dispersal linkages of the studied Chinese viruses may be modulated if other geographic Chinese viruses were to be included. However, it is worthy to restate that virus dispersals from China to Vietnam were highly certain (Fig. 4B and Supplementary Fig. S3B). Finally, we highlight the critical epidemiological role of live poultry movement, particularly cross-border poultry movement, to the transboundary dissemination of H5 HPAIVs and the necessity for preventive control against H5 HPAIV spillovers, including risks of introducing H7 HPAIVs^40^.

Even though we attempted to provide a robust elucidation of H5 HPAIVs in Vietnam, this study remains some unavoidable limitations. First, for instance, the present study investigated all Vietnamese H5 HPAIVs available via public databases but might not capture circulation of all H5 HPAIVs in the field. Second, we were unable to definitively clarify determinants for annual outbreak occurrence (i.e., low outbreak occurrence and virus detection in 2016); this may be due to interconnected effects of viral-agro-ecological factors. Thus, more systematic studies are needed to identify the determinants for outbreak occurrence of H5 HPAIVs.

In summary, the present study indicated that H5 HPAIVs persist in Vietnam and new virus strains are being introduced. During 2014–2017, clade 2.3.2.1c and 2.3.4.4 viruses were predominant and exhibited a large genetic and antigenic diversity. Considerably, these viruses were antigenically distinct from current vaccines and reference antigen used in poultry vaccination programs. Thus, this study highlighted an urgent necessity for reinforcing the provisional control measures in Vietnam including (1) strengthening surveillance for monitoring evolution of H5 HPAIVs, in particular more focus on surveillance and strict poultry movement control in border areas to prevent invasion of H5 HPAIV spillovers and (2) enhancing effectiveness of vaccination programs through updating vaccines and reference antigen to antigenically match currently circulating H5 HPAIVs.

## Materials and Methods

### Sampling, virus isolation and sequencing in our surveillance program

We conducted active surveillance of AIVs in the period 2014–2017 in representative provinces across the three geographic regions of Vietnam, North, Central and South. A total of 12,585 swabs were sampled from apparently healthy domestic birds and environment in farms, LBMs and other live poultry marketing places (LBM-like locations). The presence of AIVs was tested using M gene detection qRT-PCR. Virus isolation of M gene positive samples using chicken embryos and subtyping were performed following standard protocols^41^. Representative H5 HPAIVs based on geography and year were selected for full genome or single RNA segment sequencing using next generation or conventional Sanger sequencing, respectively^15^. H5 HPAIV strain details and sequence profiles were registered in GenBank/EMBL/DDBJ to obtain the accession numbers listed in Supplementary Table S3.

### Data collection of active and passive surveillance programs

H5 HPAI outbreak occurrence in poultry, following passive surveillance in Vietnam during the study period, was obtained from the EMPRES-i, FAO database^42^. Active surveillance program profile of AIVs in Vietnam was obtained from the annual reports of animal diseases by the national Department of Animal Health (DAH) and collated with notifiable outbreak cases as surveillance entity^5^.

Entire Vietnamese H5 HPAIV strains detected for the period 2014–2017 were obtained from the Global Initiative on Sharing All Influenza Data (GISAID) and Influenza Research Database (IRD). Background details of each strain such as infected species, sampling origins and years were available within these databases or shared by our collaborators listed in the acknowledgements section.

### Phylogenetic analysis, genotyping and molecular characterization

RNA segment sequences of the 192 representative H5 HPAIVs from our surveillance program and other H5 HPAIVs collated from GISAID and IRD were subjected to phylogenetic analyses. Nine sequence partitions derived from eight viral RNA segments (N1 and N6 NA gene segments were computed separately) were separately aligned using Muscle v3.8.3^43^. The alignments were manually inspected and poorly aligned sequence regions were removed using BioEdit v7.0.5^44^. The best-fit substitution model for each partition was selected based on the Bayesian Information Criterion using jModelTest v2.1.10^45^. Supplementary Table S4 summarizes sequence partition and modeled phylogenetic parameters with general time-reversible (GTR) substitution model following a gamma distribution. An ML tree for each partition was constructed using RAxML v8.2.4^46^ with a resampling process of 1,000 rapid replicates. All ML trees were rooted with a corresponding gene segment of the Gs/GD precursor as expected topology. Phylogenetic trees were annotated and visualized using Figtree v1.4.3^47^ or ggtree package^48^ implemented in R v3.4.4^49^. Clade assignments and genotyping were determined from common phylogenetic relationships observed among the eight gene segments, following the standard criteria of WHO/OIE/FAO nomenclature and previous classification reports^7–10,21^.

### HI assay, antigenic cartography and antibody assessment

MDCK plaque-cloned viruses representing each H5 HA clade assignment were obtained for antigenic analysis. Antigenicity of these cloned H5 HPAIVs within and between clade assignments was determined by cross-HI test using a panel of chicken antisera as previously described^29,41^. To obtain an overall picture of antigenic evolution, the contemporary Vietnamese viruses, progenitor viruses including A/Hong Kong/483/1997 (H5N1) clade 0 virus; A/tern/South Africa/1961 (H5N3) reference virus; and the A/chicken/Scotland/1959 (H5N1) reference antigen were included for antigenic cartography. Intentionally, all the viruses were formalin-inactivated as a comparable form to the commercial reference antigen purchased from APHA Scientific, UK. These antigens cross-reacted with several reference laboratory antisera^29,41^ and antisera collected from two chickens and two ducks immunized with commercial clade 1 and 2.3.2 vaccines under laboratory condition. Each chicken and duck was intramuscularly immunized with 0.5 mL clade 1 or 2.3.2 vaccines as manufacturer’s instruction. The resulting data containing cross-HI titers was loaded to the web-based software (http://www.antigenic-cartography.org/) to obtain x/y coordinates of each antiserum and antigen. Antigenic cartography was then visualized using R v3.4.4.

Field antisera from chickens and ducks vaccinated with commercial vaccines in Vietnam were collected to test antibody titers. Vaccination status of individual birds was confirmed by farmers and local veterinarians, as described in Supplementary Table 7. Field antisera were transferred to Japan and tested using the HI test as described above.

### Spatiotemporal phylodynamic analysis using Bayesian method

A Bayesian method using BEAST v1.8.4^50^ was performed to explore spatiotemporal phylodynamics of two predominant clade 2.3.2.1c and 2.3.4.4 H5 HPAIVs since their first detection in Vietnam to 2017 (from 2012 for clade 2.3.2.1c viruses and 2013 for clade 2.3.4.4 viruses, respectively^2,9,10^). HA sequences of H5 HPAIV strains in Cambodia, Laos and southern neighboring Chinese provinces were combined with our HA sequence partition of each clade. The sampling dates and locations for each sequence were provided to BEAST to estimate space-time divergence. The lognormal uncorrelated relaxed clock model, the exponential growth tree prior and other parameters were used and described in Supplementary Table S4. For each clade dataset, the Bayesian Markov Chain Monte Carlo (MCMC) chains (one chain per dataset) were run for 90 million states, sampled every 5,000 iterations in a web-based CIPRES Science Gateway v3.3^51^. Convergence of the chains was assessed using Tracer 1.7^52^. Maximum clade credibility (MCC) trees were summarized using Tree Annotator v1.8.4; with 10% of the first sampled trees removed. Each MCMC analysis was independently performed at least twice and compared to ensure reproducibility. The final MCC trees containing space-time divergence were converted to a keyhole markup language file using SPREAD v1.0.7^53^. Then, spatial-temporal dispersal linkages of the two predominant H5 HPAIV population were visualized using several spatial visualization packages (ggplot2, ggmap, rgeos, sp, and maptools) implemented in R v3.4.4.

### Ethical approval and informed consent

All the viral experimentation was conducted in a biosafety level 3 facility in our affiliation (approval number: 13-0108). Animal experiments for preparing laboratory antisera (approval number: 13-0093) were performed according to the guidelines of the Institutional Animal Care and Use Committee of the Faculty of Veterinary Medicine, Hokkaido University, which is accredited by the Association for Assessment and Accreditation of Laboratory Animal Care International. Our active surveillance program was authorized by the Vietnam DAH, Hokkaido University, Agency for Medical Research and Development, Japan (AMED) and the Ministry of Agriculture and Forestry and Fisheries of Japan (MAFF). Informed consent of farmers, sellers and other participants for our sampling was obtained.

## Supplementary information

Supplementary Figures

Supplementary Tables

## Data Availability

The data that support the findings of this study are included in this published article and its supplementary materials (Supplementary Figs S1–S4 and Supplementary Tables S1–S8).
