# Supplementary material for: A systematic study towards evolutionary and epidemiological dynamics of currently predominant H5 highly pathogenic avian influenza viruses in Vietnam
Source: Sci Rep. 2019 May 22;9:7723. doi: 10.1038/s41598-019-42638-4 (PMC6531488; doi:10.1038/s41598-019-42638-4)
Supplement: Supplementary file 1 — Supplementary Figures [file 41598_2019_42638_MOESM1_ESM.pdf]

Supplementary Information

A systematic study towards evolutionary and epidemiological dynamics of currently predominant H5 highly pathogenic avian influenza viruses in Vietnam

Lam Thanh Nguyen[1,2], Simon M. Firestone[3], Mark A. Stevenson[3*], Neil D. Young[3], Les Sims[4], Duc Huy Chu[5], Tien Ngoc Nguyen[5], Long Van Nguyen[5], Tung Thanh Le[5], Hung Van Nguyen[5], Hung Nam Nguyen[5], Tien Ngoc Tien[6], Tho Dang Nguyen[7], Bich Ngoc Tran[2], Keita Matsuno[1,8], Masatoshi Okamatsu[1], Hiroshi Kida[8,9], Yoshihiro Sakoda[1,8*]

[1] Laboratory of Microbiology, Faculty of Veterinary Medicine, Hokkaido University, Sapporo, Hokkaido, 060-0818, Japan

[2] Department of Veterinary Medicine, College of Agriculture, Can Tho University, Can Tho, Vietnam

[3] Melbourne Veterinary School, Faculty of Veterinary and Agricultural Sciences, The University of Melbourne, Parkville, Victoria, 3010, Australia

[4] Asia Pacific Veterinary Information Services, Montmorency, Victoria, 3094, Australia

[5] Department and Sub-Departments of Animal Health, Ministry of Agriculture and Rural Development, Ha Noi, Vietnam

[6] Regional Animal Health Office VII, Department of Animal Health, Ministry of Agriculture and Rural Development, Can Tho, Vietnam

[7] National Center for Veterinary Diagnostics, Department of Animal Health, Ministry of Agriculture and Rural Development, Ha Noi, Vietnam

[8] Global Institution for Collaborative Research and Education, Hokkaido University, Sapporo, Hokkaido, 001-0020, Japan

[9] Research Center for Zoonosis Control, Hokkaido University, Sapporo, Hokkaido 001-0020, Japan

Supplementary figure legends

Supplementary Figure S1. Summary statistics of different species infected with H5 HPAIVs (excluding our surveillance program), obtained from GISAID and/or IRD. Numbers and sizes of each circle are the sums of infected individuals.

Supplementary Figure S2. Maximum likelihood phylogenetic trees of eight gene segments of H5 HPAIVs. HA **(A)**; N1 NA **(B)**; N6 NA **(C)**; PB2 **(D)**; PB1 **(E)**; PA **(F)**; NP (**G)**; M **(H)**; and NS **(I)**. Newly identified cluster(s) are highlighted in the red branch. Bootstrap values are shown for key nodes.

Supplementary Figure S3. Bayesian phylogenetic trees of H5 HA gene segments of clade 2.3.2.1c **(A)** and 2.3.4.4 **(B)** H5 HPAIVs detected in Vietnam, Cambodia, Laos and southern bordering provinces of China for the period 2012–2017. Posterior probabilities are shown for key nodes.

Supplementary Figure S4. Spatiotemporal phylodynamics of two predominant clade 2.3.2.1c **(A)** and 2.3.4.4 **(B)** H5 HPAIVs in Vietnam and other neighboring countries for the period 2012–2013. Epidemiological dispersal linkages from one location to another are indicated by arrows.

S. Fig. S1

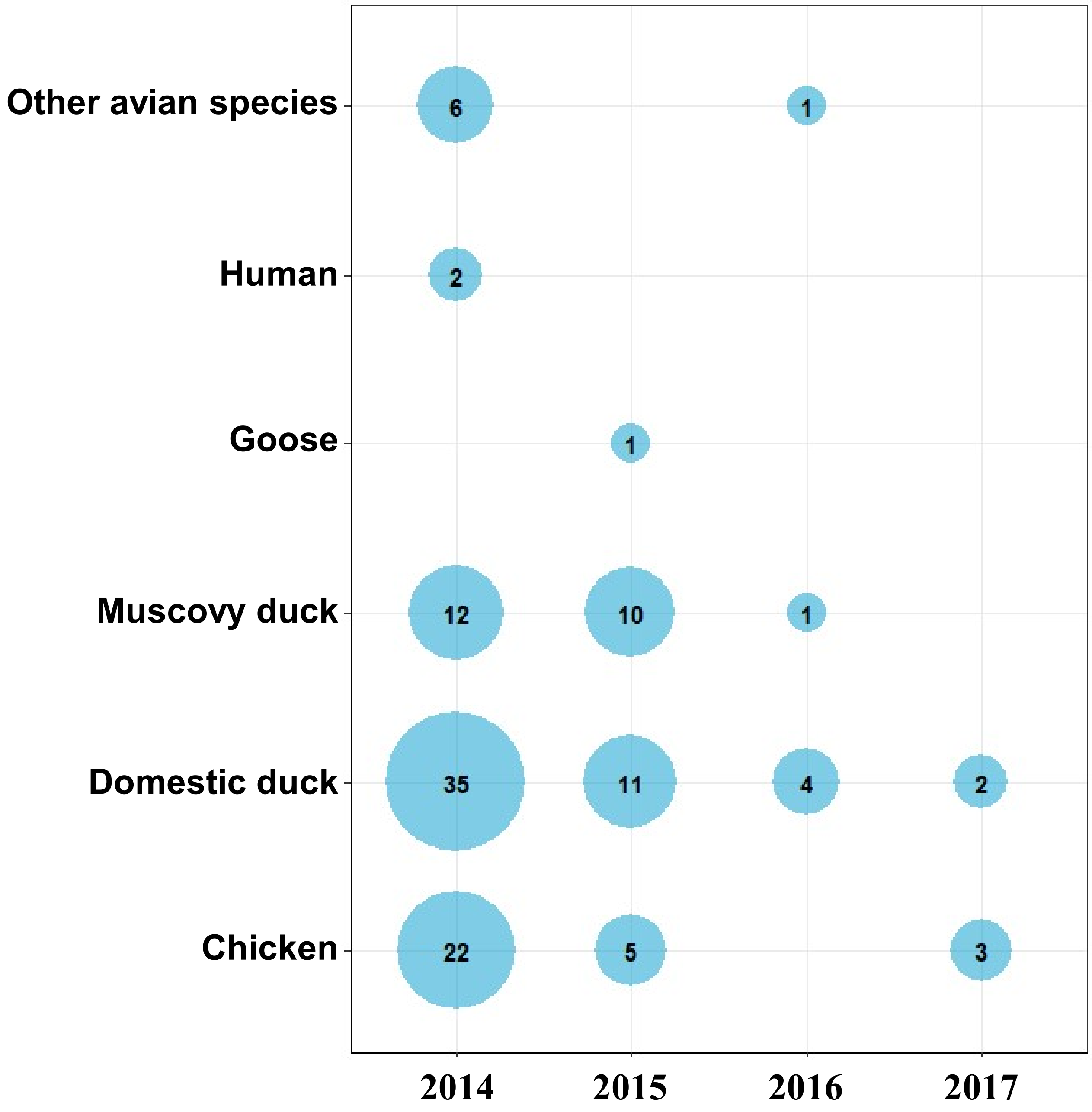

Other avian species
Human
Goose
Muscovy duck
Domestic duck
Chicken
6
1
2
1
12
10
1
35
11
4
2
22
5
3
2014
2015
2016
2017

S. Fig. S2-A

HA segment

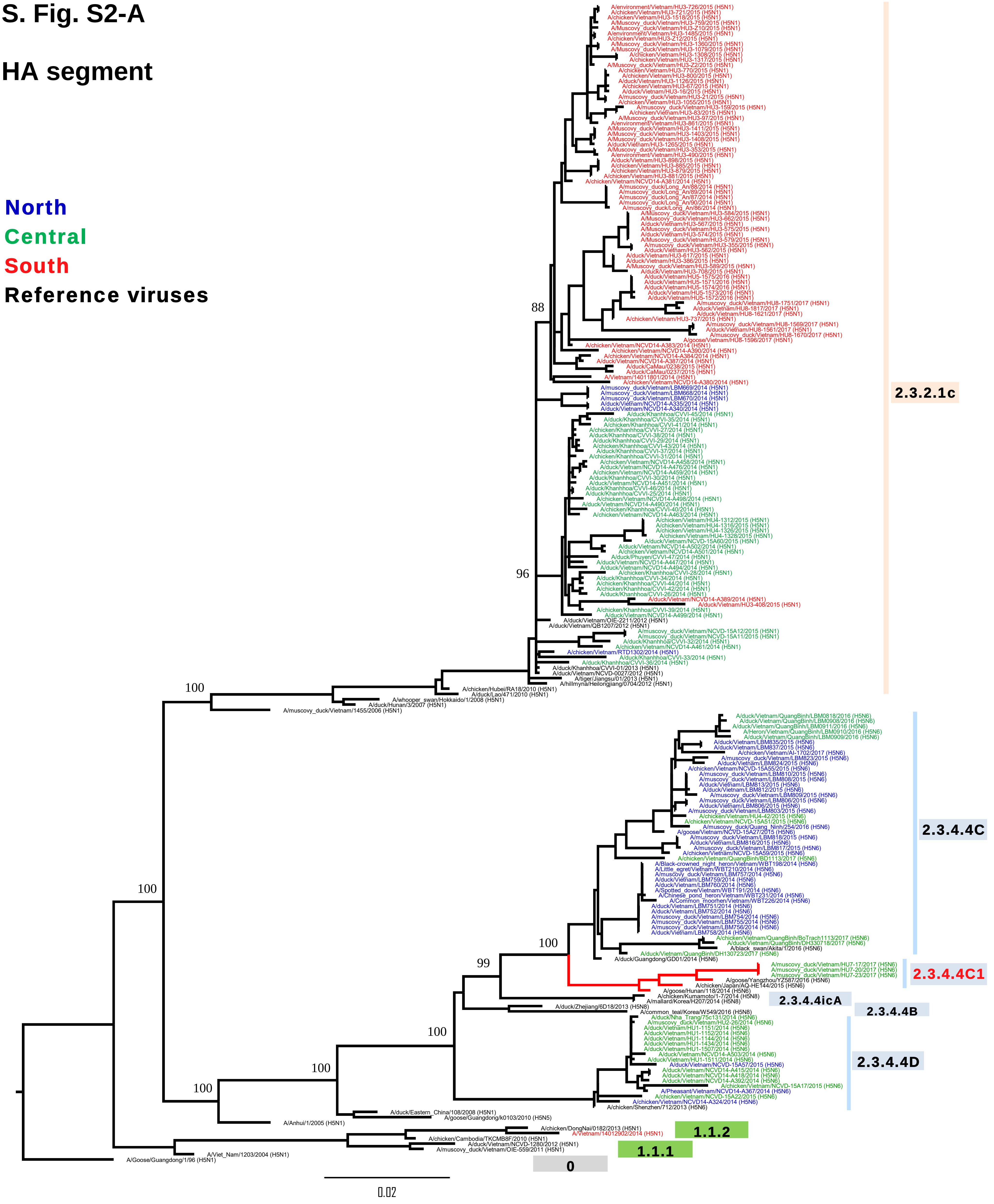

S. Fig. S2-B

N1 NA segment

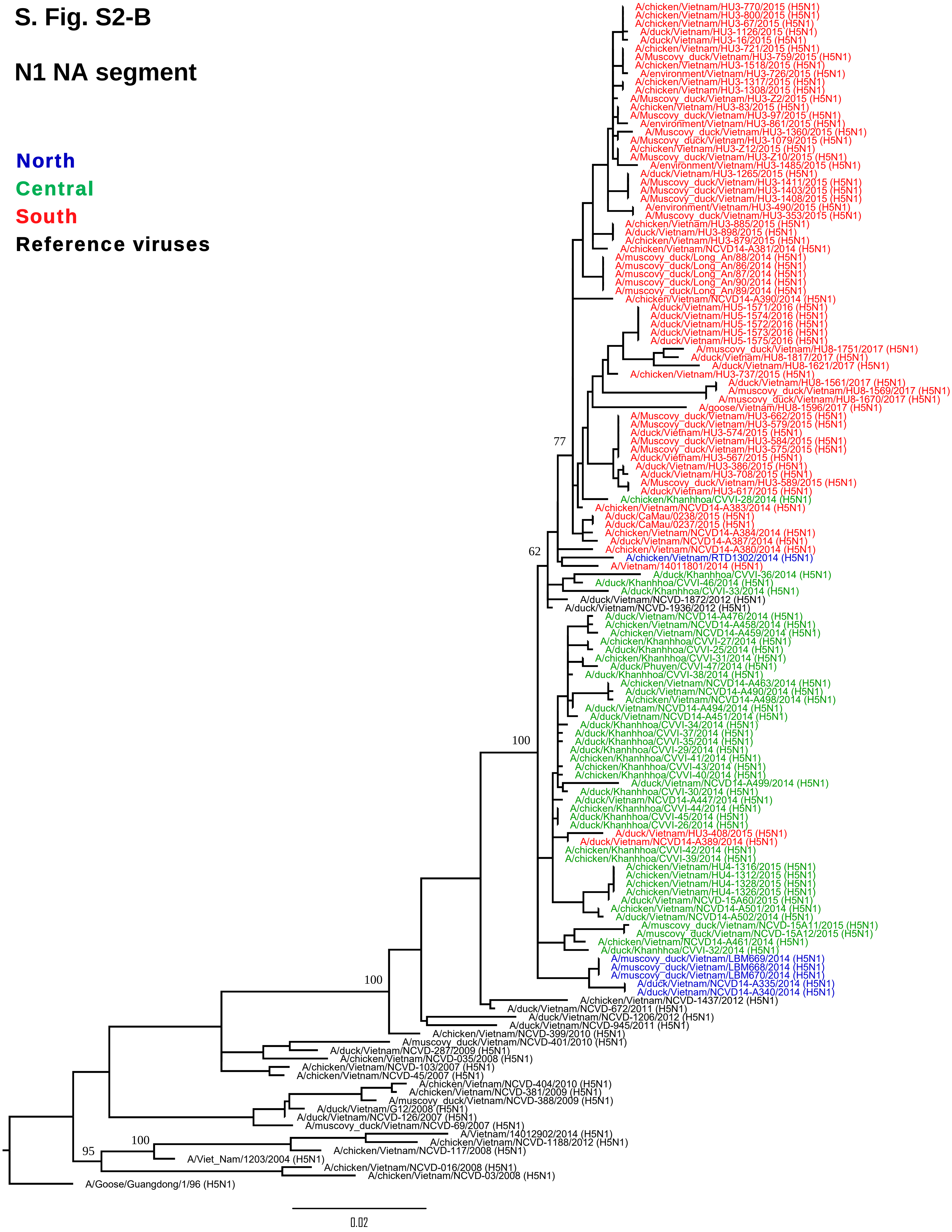

S. Fig. S2-C

N6 NA segment

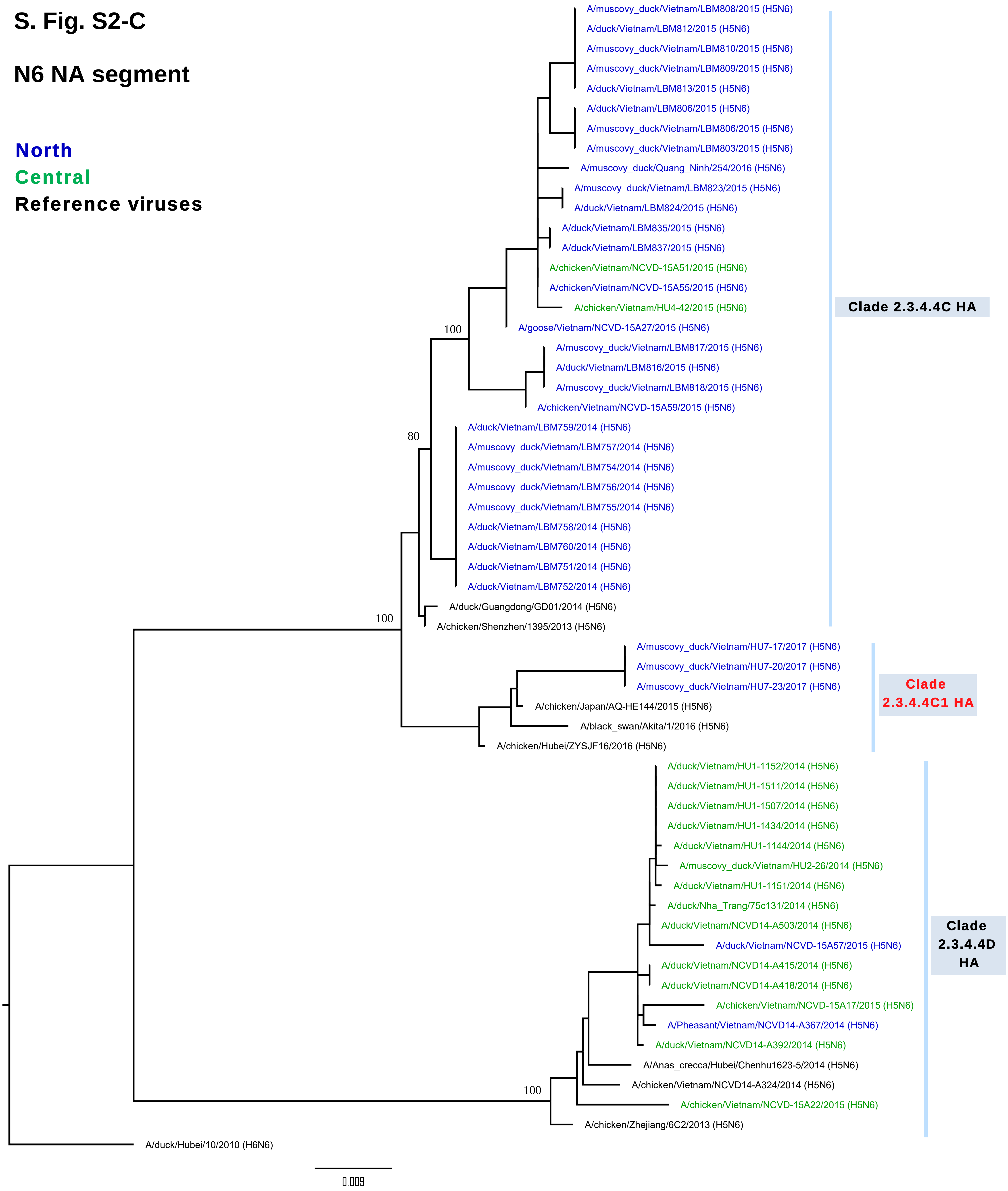

S. Fig. S2-D

PB2 segment

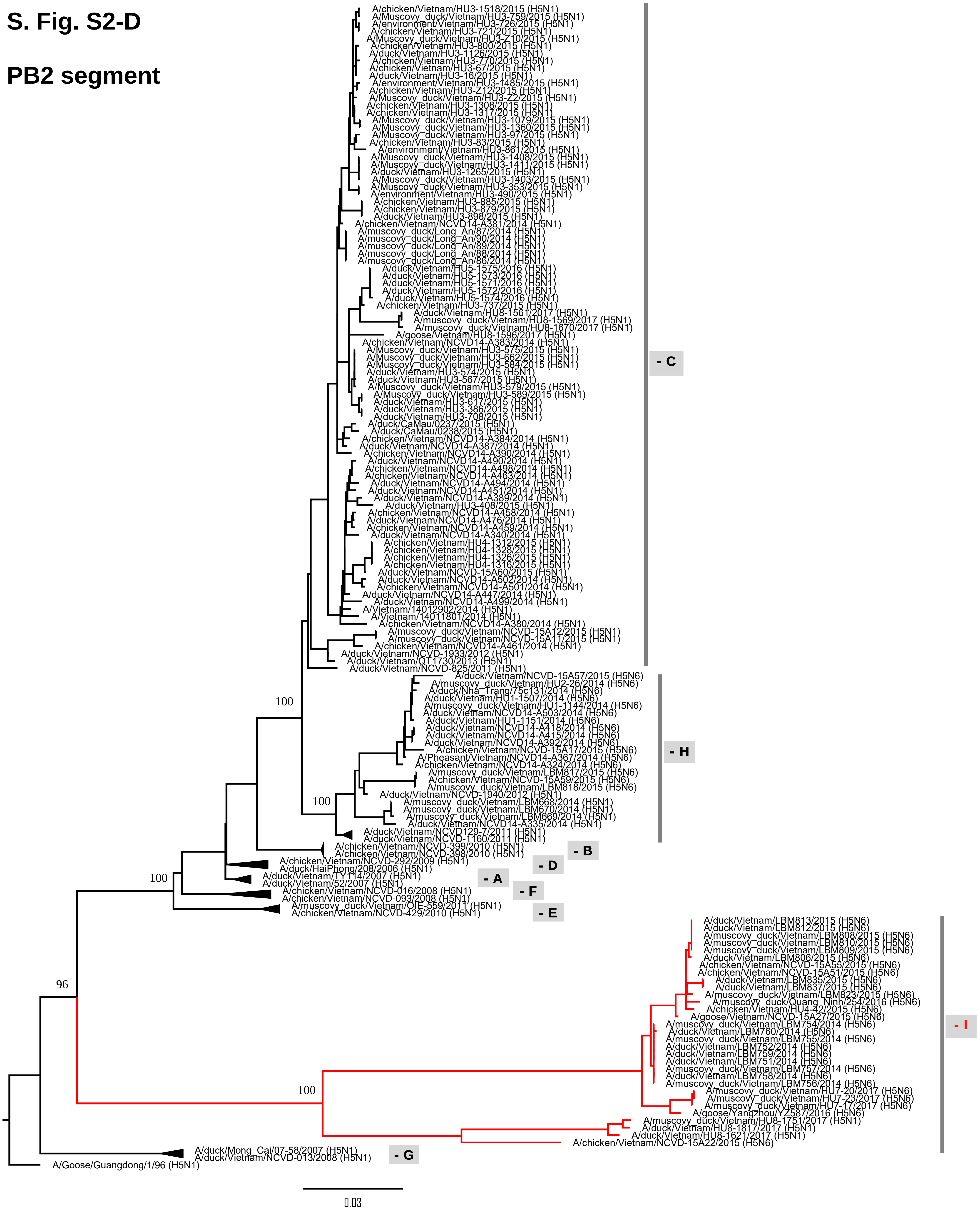

S. Fig. S2-E

PB1 segment

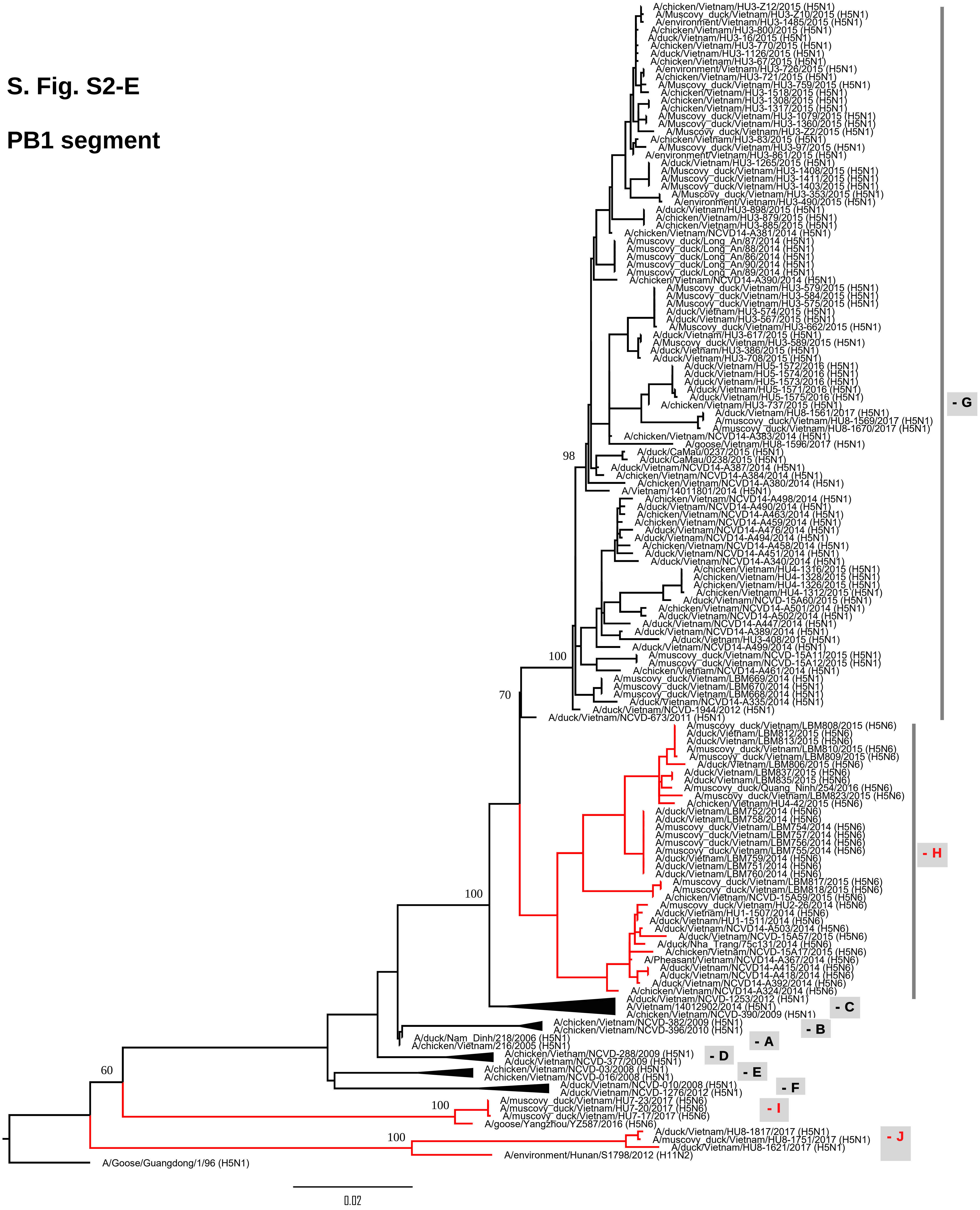

S. Fig. S2-F

PA segment

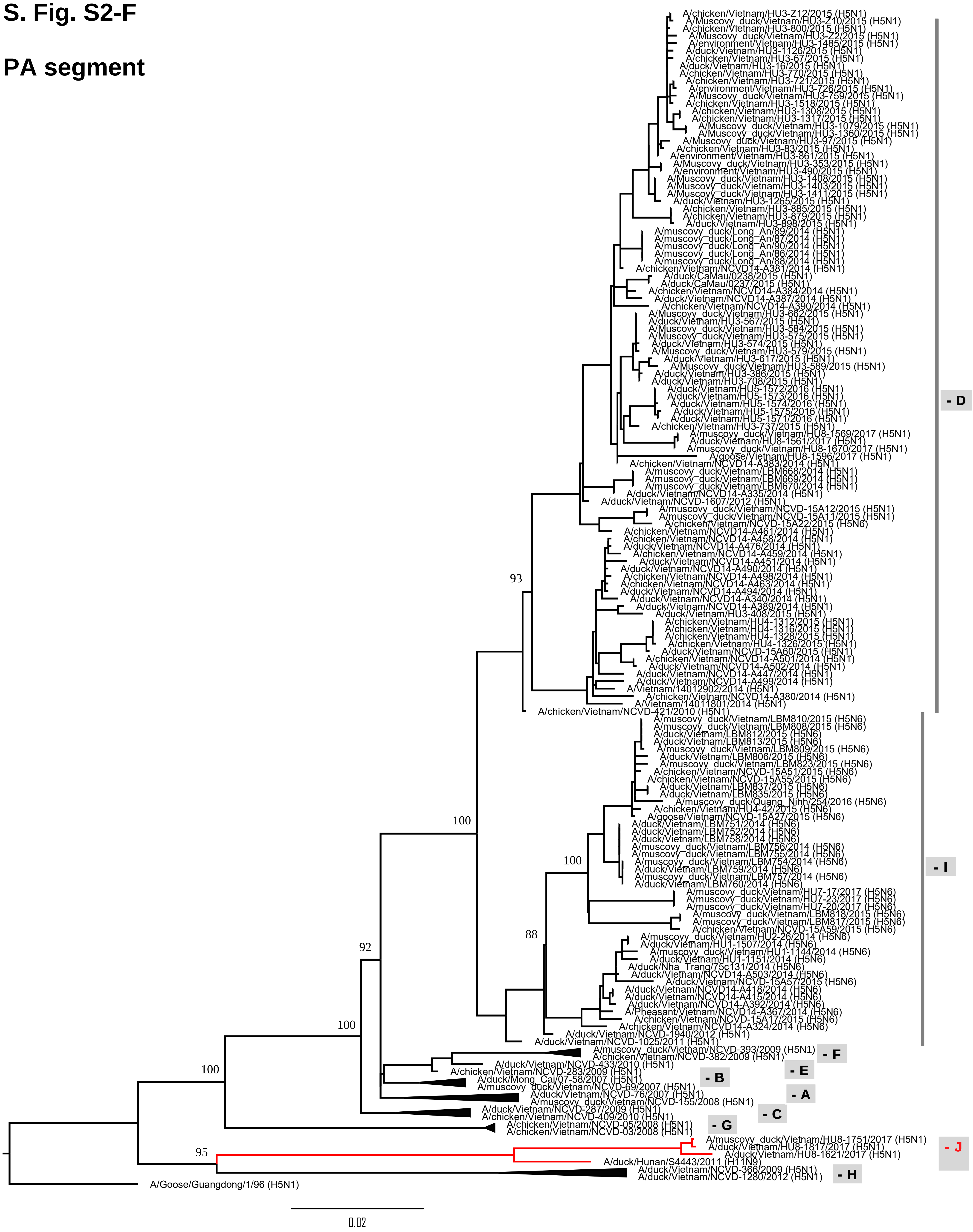

S. Fig. S2-G

NP segment

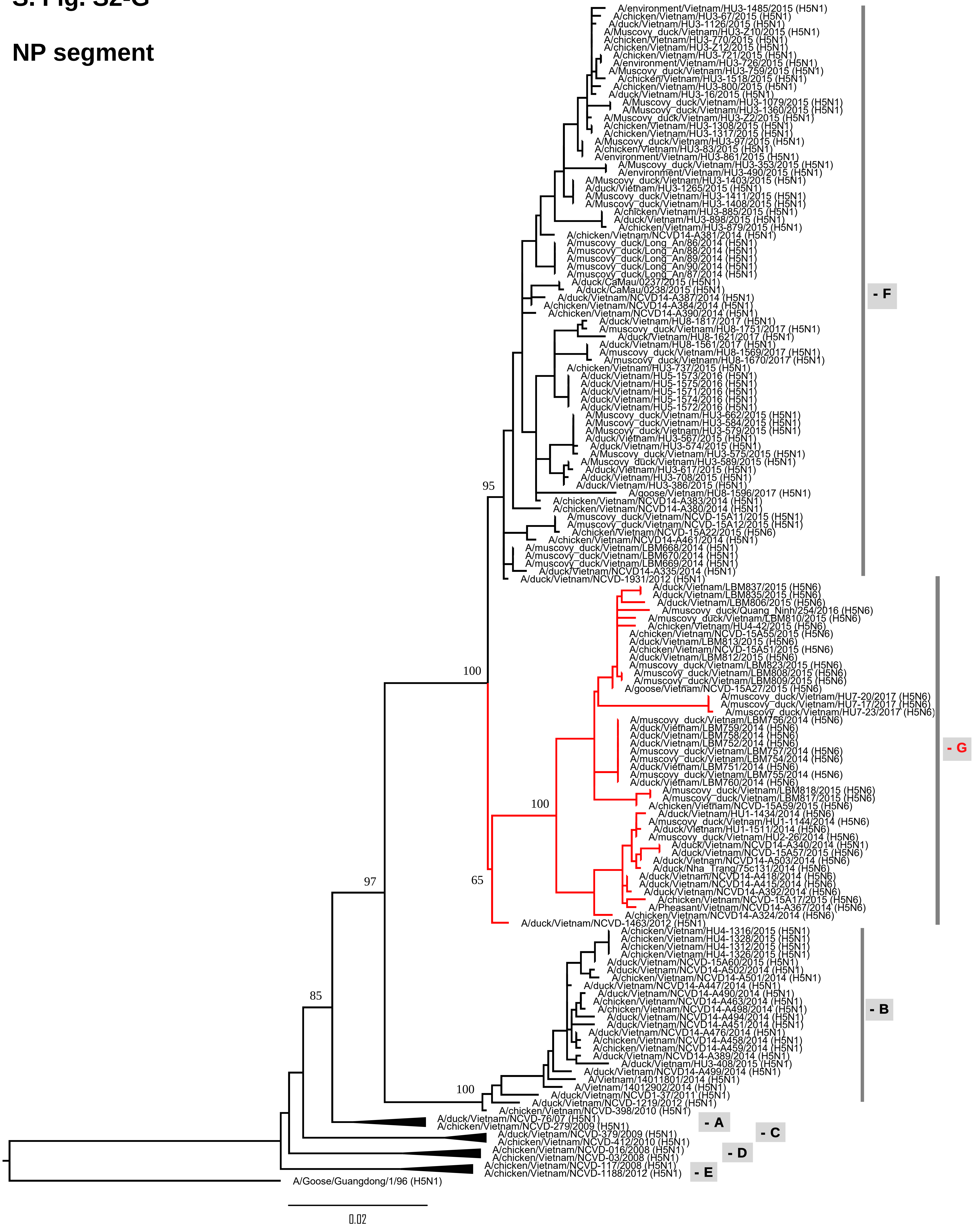

S. Fig. S2-H

M segment

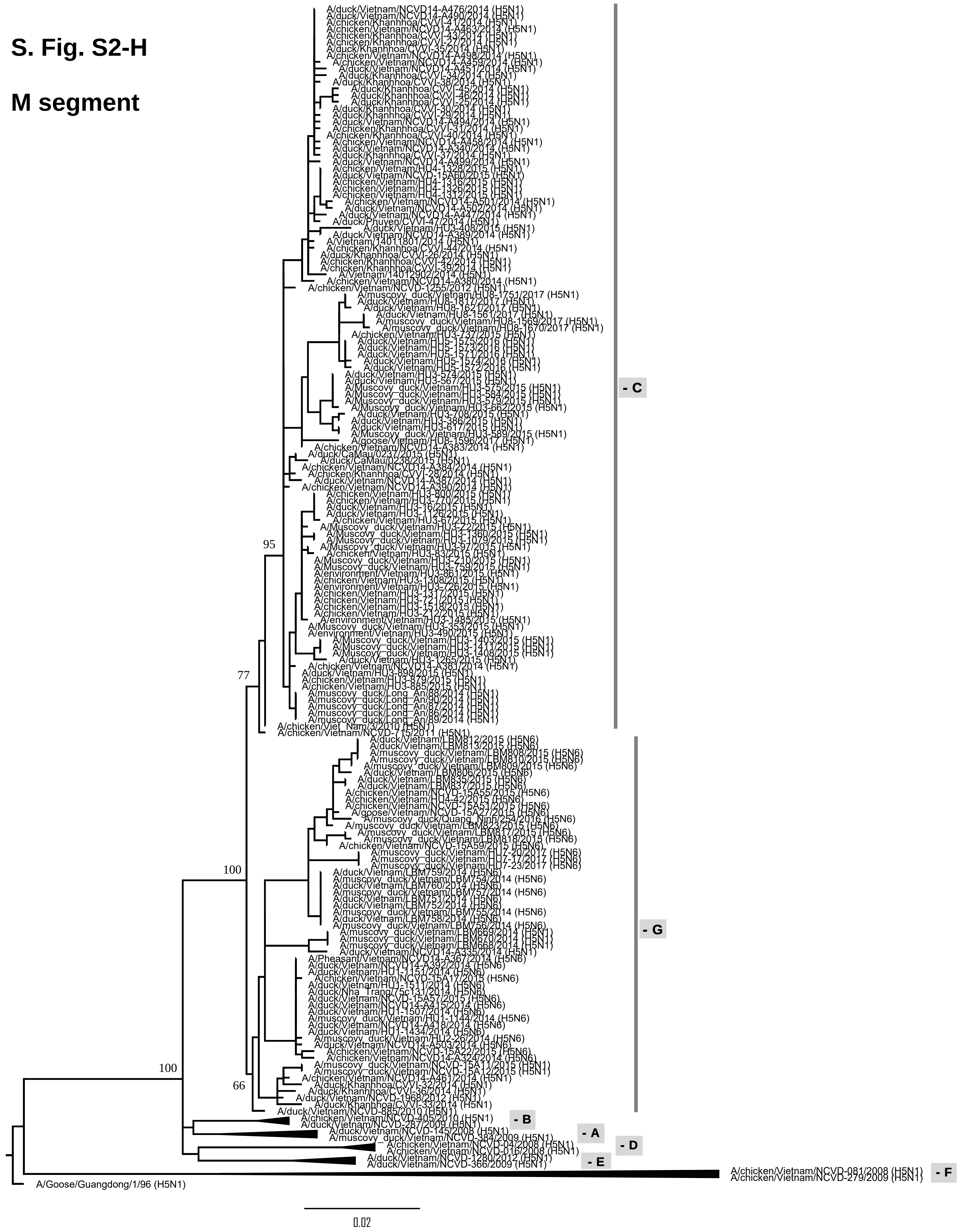

S. Fig. S2-I

NS segment

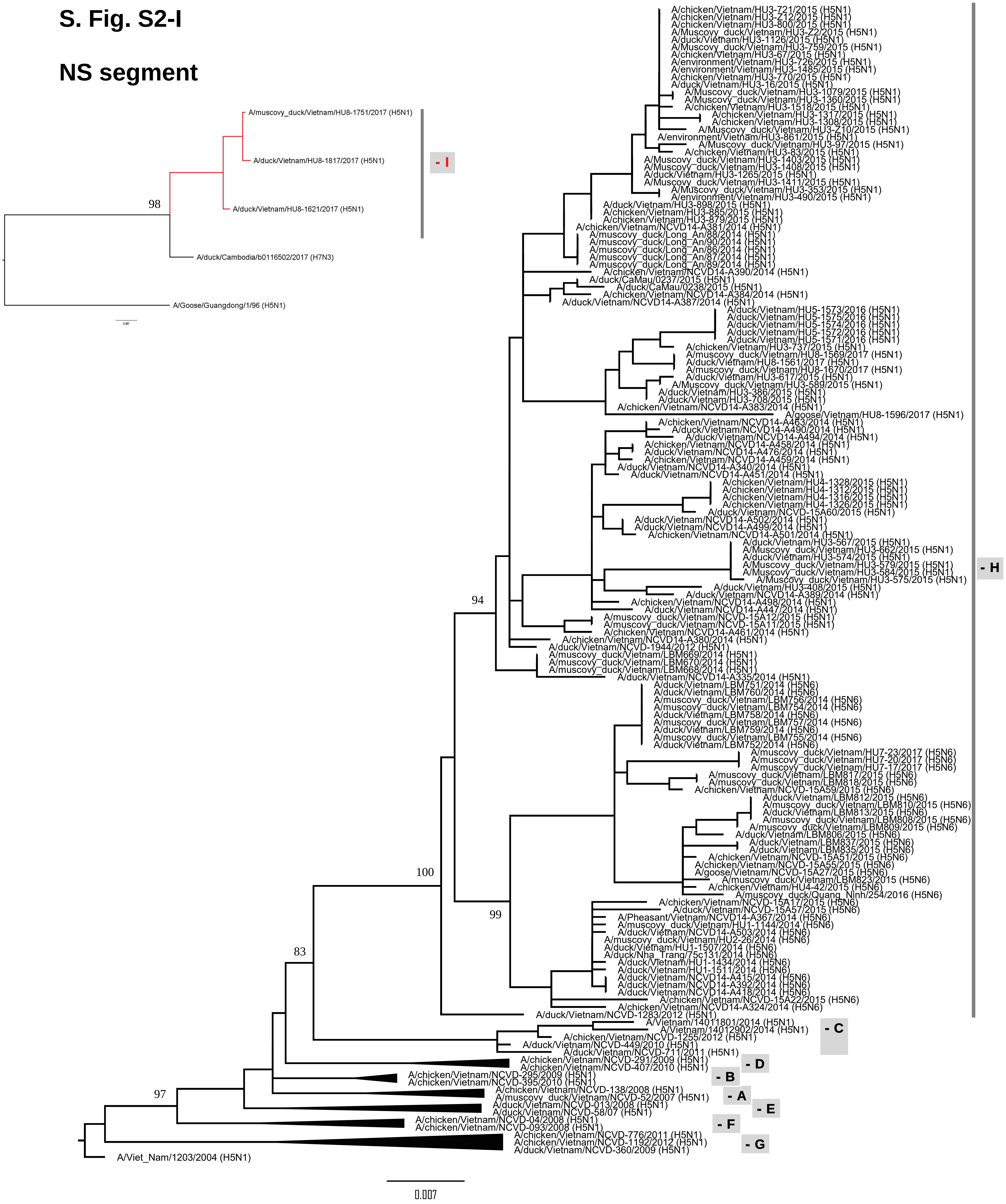

S. Fig. S3-A

Clade 2.3.2.1c H5 HA segment

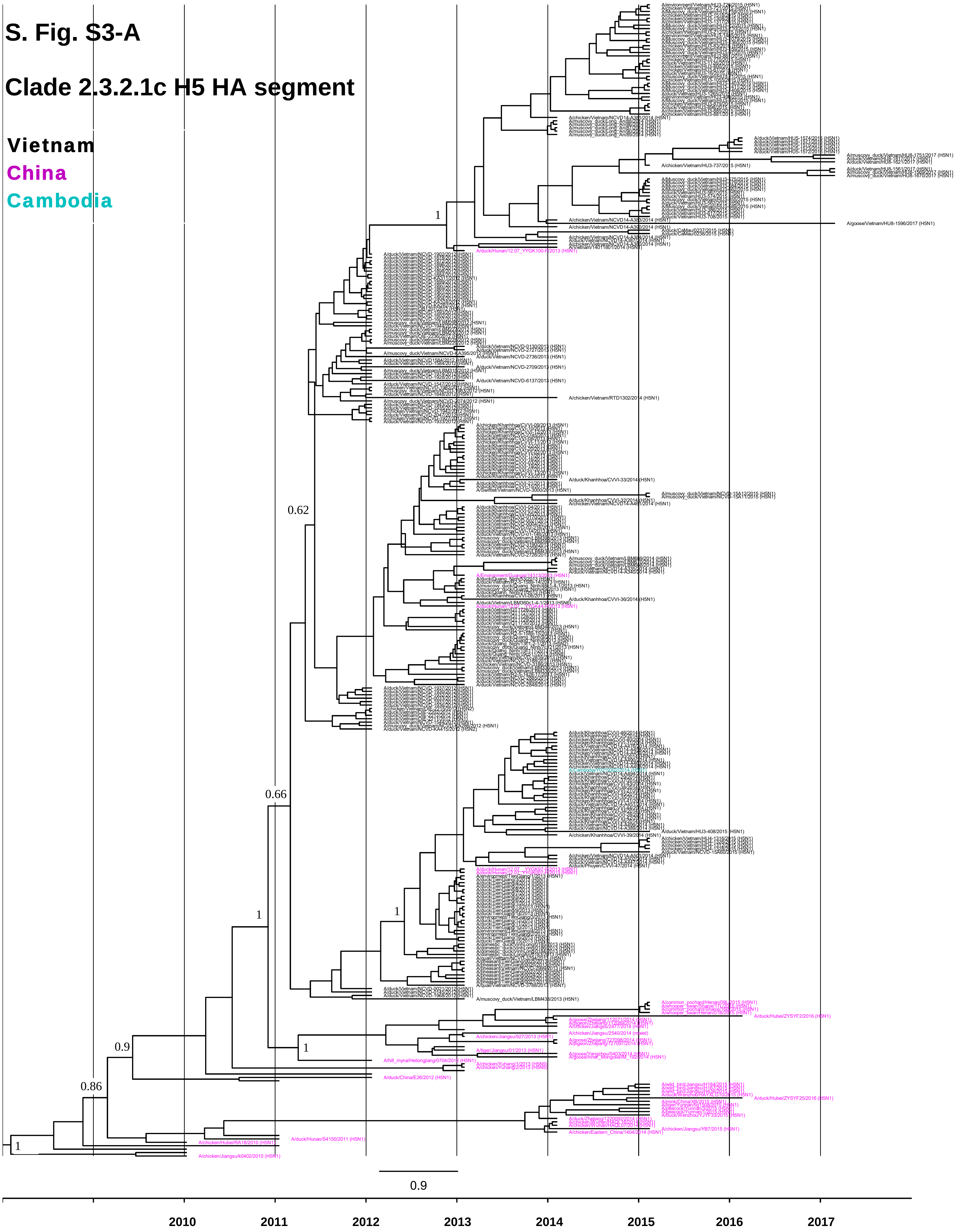

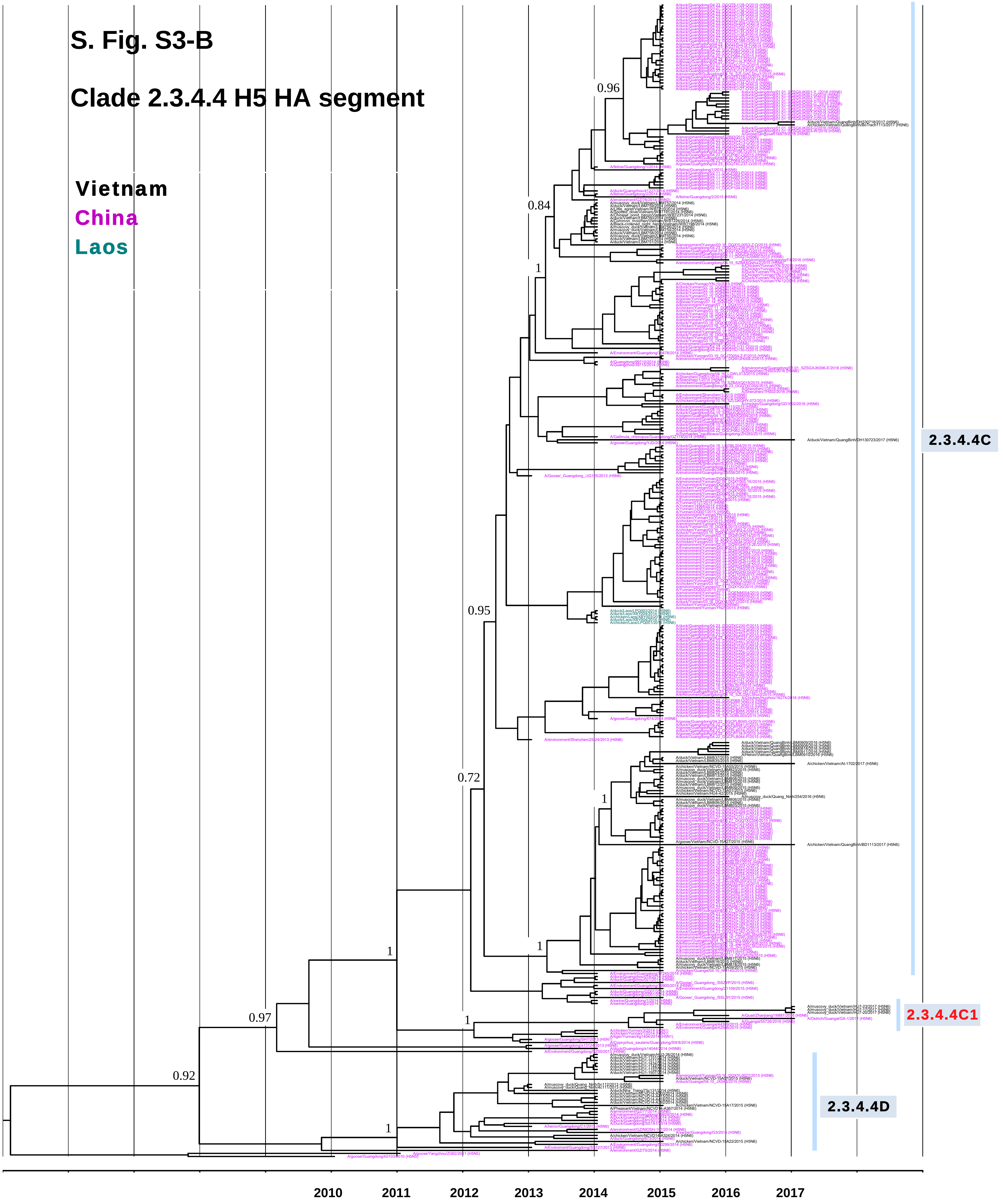
S. Fig. S3-B
Clade 2.3.4.4 H5 HA segment
Vietnam
China
Laos
2.3.4.4C
2.3.4.4C1
2.3.4.4D
0.96
0.84
1
0.95
0.72
1
1
1
0.97
1
0.92
1
2010
2011
2012
2013
2014
2015
2016
2017

S. Fig. S4

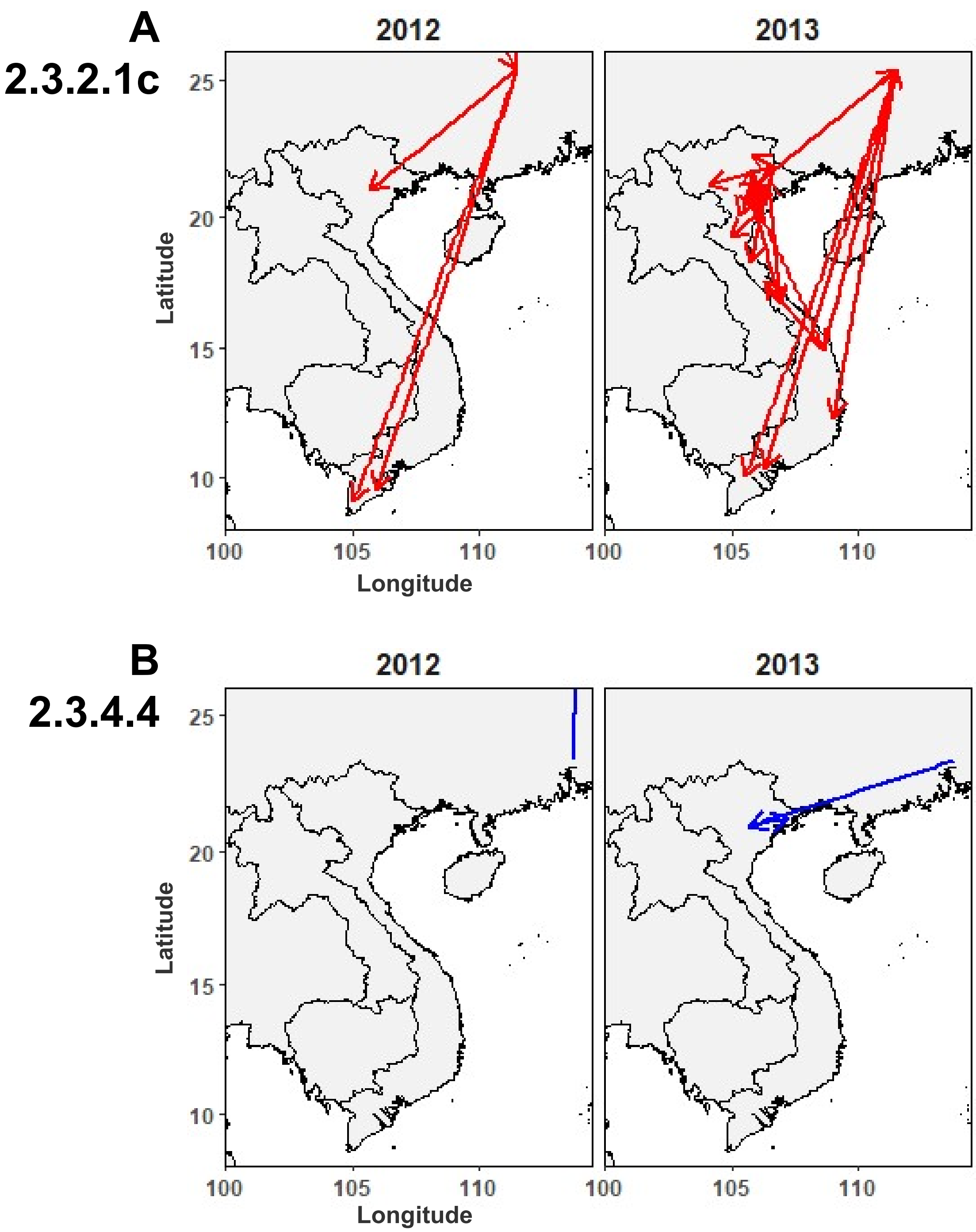
A
2.3.2.1c
2012
2013
25
20
15
10
Latitude
100
105
110
Longitude
B
2.3.4.4
